# RUNX2 recruits the NuRD(MTA1)/CRL4B complex to promote breast cancer progression and bone metastasis

**DOI:** 10.1038/s41418-022-01010-2

**Published:** 2022-05-09

**Authors:** Xin Yin, Xu Teng, Tianyu Ma, Tianshu Yang, Jingyao Zhang, Miaomiao Huo, Wei Liu, Yunkai Yang, Baowen Yuan, Hefen Yu, Wei Huang, Yan Wang

**Affiliations:** 1 Beijing Key Laboratory of Cancer Invasion and Metastasis Research, Department of Biochemistry and Molecular Biology, School of Basic Medical Sciences, Capital Medical University, Beijing, 100069 China; 2 Key Laboratory of Cancer and Microbiome, State Key Laboratory of Molecular Oncology, National Cancer Center/National Clinical Research Center for Cancer/Cancer Hospital, Chinese Academy of Medical Sciences and Peking Union Medical College, Beijing, 100021 China; 3 Key Laboratory of Immune Microenvironment and Disease (Ministry of Education), Department of Biochemistry and Molecular Biology, School of Basic Medical Sciences, Tianjin Medical University, Tianjin, 300070 China

**Keywords:** Cancer, Epigenetics, Metastasis

## Abstract

Runt-related transcription factor 2 (RUNX2) is an osteogenesis-related transcription factor that has emerged as a prominent transcription repressing factor in carcinogenesis. However, the role of RUNX2 in breast cancer metastasis remains poorly understood. Here, we show that RUNX2 recruits the metastasis-associated 1 (MTA1)/NuRD and the Cullin 4B (CUL4B)-Ring E3 ligase (CRL4B) complex to form a transcriptional-repressive complex, which catalyzes the histone deacetylation and ubiquitylation. Genome-wide analysis of the RUNX2/NuRD(MTA1)/CRL4B complex targets identified a cohort of genes including peroxisome proliferator-activated receptor alpha (PPARα) and superoxide dismutase 2 (SOD2), which are critically involved in cell growth, epithelial-to-mesenchymal transition (EMT) and invasion. We demonstrate that the RUNX2/NuRD(MTA1)/CRL4B complex promotes the proliferation, invasion, tumorigenesis, bone metastasis, cancer stemness of breast cancer in vitro and in vivo. Strikingly, RUNX2 expression is upregulated in multiple human carcinomas, including breast cancer. Our study suggests that RUNX2 is a promising potential target for the future treatment strategies of breast cancer.

## Introduction

Breast cancer, the most common cancer among women worldwide, is the leading cause of cancer-related deaths in women [1, 2]. Approximately 90% of deaths in patients with breast cancer are attributed to metastasis. Bone metastasis is the most common distant metastasis site, accounting for almost 80% of metastatic cases [3]. During metastatic dissemination, breast cancer cells from the primary tumor must first undergo epithelial-to-mesenchymal transition (EMT) to invade the surrounding tissue, enter the microvasculature (intravasation) of the blood, and finally settle in the bone tissue [4, 5]. Bone metastasis is mediated by the interaction of breast cancer cells with osteoblasts and osteoclasts and involve aberrant bone resorption, which promotes the formation of a premetastatic niche [6].

Runt-related transcription factor 2 (RUNX2), a transcription factor involved in bone development, and its expression is upregulated in preosteoblasts to regulate the maturation and proliferation of osteoblast progenitors [7, 8]. Recent studies have shown that RUNX2 is overexpressed in several tumors and is associated with malignant progression and poor outcomes, such as osteosarcoma, prostate cancer, and breast cancer [9–13]. RUNX2 can promote prostate tumor growth and metastasis [14]. In breast cancer, RUNX2 has been found to promote breast cancer progression by driving EMT-like change and DNA damage [11, 15]. Moreover, RUNX2 can interact directly with estrogen receptors and inhibit cellular apoptosis and differentiation [16, 17]. It is suggested that RUNX2 might play a critical role in building a bone microenvironment to facilitate cancer cell to bone, however, the molecular mechanism needs to be further investigated.

The nucleosome remodeling and deacetylase (NuRD) complex is a chromatin-remodeling complex with important transcription, carcinogenesis, cell cycle progression, and genomic stability functions [18]. Of all the NuRD complex subunits, the MTA family members, involved in the deacetylation of histones, may be the most promising modulators in cancer development [19]. The MTA family shares many similar characteristics, but each of these three members, MTA1, MTA2, and MTA3, exhibit significant differences in cancer progression and metastasis [20]. According to previous studies, MTA1 repressed SMAD7 transcription to activate TGFβ signaling and assisted carcinogenesis and metastasis [21]. MTA1 is also highly expressed in metastatic tumors and bone metastatic lesions [22]. MTA1 might target CTSB to mediate cell invasion and the development of bone metastasis in prostate cancer [23]. However, the role of MTA1 in breast cancer related to bone metastasis requires further analysis.

Cullin4B (CUL4B) is a scaffold protein of the CUL4B-Ring E3 ligase (CRL4B) complex. Our previous study demonstrated that the CRL4B complex could coordinate with SIRT1 to regulate pancreatic cancer metastasis and stem cell properties to promote tumorigenesis [24]. Similarly, we found that CRL4B interacted with multiple histone deacetylase (HDAC)-containing co-repressor complexes, such as NuRD(MTA1) complex, promoting the EMT process and tumorigenesis in breast cancer [25]. In addition, another study showed that CUL4B promoted the gastric cancer EMT process by upregulating HER2 [26]. However, the role of CUL4B in breast cancer bone metastasis has not yet been explored.

In this study, we investigated the potential role of RUNX2 in the development of breast cancer and bone metastasis. Here, we confirmed that RUNX2 could recruit the NuRD(MTA1)/CRL4B complex to induce cell proliferation, invasion, tumorigenesis, and bone metastasis, as well as cancer stemness. Our results indicate that RUNX2 is a promising potential target for the future treatment strategies of breast cancer.

## Materials and methods

### Antibodies and reagents

The antibodies used in this study were: anti-FLAG (F1408, Sigma–Aldrich, St. Louis, MO, USA), anti-RUNX2 (ab236639, Abcam, Cambridge, UK), anti-MTA1 (sc-10813, Santa Cruz Biotechnology, Dallas, TX, USA), anti-MTA2 (ab50209, Abcam), anti-MTA3 (IM1012, Millipore, Billerica, MA, USA), anti-HADC1 (H3284, Sigma–Aldrich), anti-HADC2 (H3159, Sigma–Aldrich), anti-RbAp46/48 (R3779, Sigma–Aldrich), anti-MBD3 (sc-271521, Santa Cruz Biotechnology), anti-DDB1 (sc-25367, Santa Cruz Biotechnology), anti-CUL4B (C9995, Sigma–Aldrich), anti-ROC1 (ab2977, Abcam), anti-E-cadherin (610181, BD Biosciences, Franklin Lakes, NJ, USA), anti-α-catenin (610193, BD Bioscience), anti-γ-catenin (610253, BD Bioscience), anti-Fibronectin (F3648, Sigma–Aldrich), anti-N-cadherin (610920, BD Bioscience), anti-Vimentin (V6630, Sigma–Aldrich), anti-VEGFA (ER30607, HUABIO, Hangzhou, China), anti-PTH1R (EM1709-55, HUABIO), anti-IL-8 (R1511-15, HUABIO), anti-MMP3 (#14351, Cell Signaling Technology, Massachusetts, USA), anti-SOX2 (ab59776, Abcam), anti-NANOG (ab109250, Abcam), anti-OCT4 (ab19857, Abcam), anti-KLF4 (12173, Cell Signaling Technology), anti-PPARα (66826-1-lg, Proteintech, Rocky Hill, NJ, USA), anti-SOD2 (D3X8F, Cell Signaling Technology), and anti-β-actin (A1978, Sigma–Aldrich). Dynabeads protein A/G and Glutathione-Sepharose 4B beads were purchased from Invitrogen (Thermo Fisher Scientific, Waltham, MA, USA) and Gene Pharma Co., respectively. The protease inhibitor cocktail was purchased from Roche Applied Science. The siRNAs for RUNX1, RUNX2, RUNX3, SOD2, and PPARα were obtained from GenePharma (Shanghai, China). Short hairpin RNAs (shRNAs) were obtained from Shanghai GenePharma.

### Cell culture and transfection

All cell lines used in this study were obtained from the American Type Culture Collection, tested and authenticated by STR profiling. Human triple negative breast cancer MDA-MB-231 and SUM159 cells were cultured in Dulbecco’s modified Eagle’s medium (DMEM) and RPMI1640 medium supplemented with 10% fetal bovine serum (FBS), respectively. The mouse preosteoblastic cell line MC3T3-E1 was cultured in minimum essential medium-alpha (MEM, Thermo Fisher Scientific). Mouse mononuclear macrophage RAW264.7 cells were cultured in DMEM with 10% FBS. Cells were maintained in a sterile, humidified incubator with 5% CO_2_ at 37 °C. siRNAs were transfected into cells using Lipofectamine RNAiMAX Reagent (Invitrogen, Carlsbad, CA, USA, USA) according to the manufacturer’s instructions. For RNAi experiment, each gene was tested using at least three independent siRNA/shRNA sequences, and the one with the highest efficiency was used. The siRNA and shRNA sequences used were listed in Supplementary Table S1–2.

### Immunopurification and mass spectrometry

A FLAG-tagged RUNX2 plasmid was transfected into MDA-MB-231 cells, which were harvested 48 h later. Anti-FLAG immune affinity columns were prepared using anti-FLAG M2 affinity gel (Sigma–Aldrich), following the manufacturer’s instructions. Cell lysates were obtained from approximately 5 × 10^8^ cells and applied to an equilibrated FLAG column of 1 mL bed volume to allow for the adsorption of the protein complex to the column resin. After binding, the column was washed with cold BC500 buffer. Then, FLAG peptide (0.2 mg*/*mL, Sigma–Aldrich) was added to the column to elute the FLAG protein complex according to the reagent’s introduction. Fractions of the bed volume were collected and resolved on SDS-PAGE and silver stained, and gel bands were excised and subjected to LC-MS/MS sequencing and data analysis.

### Immunoprecipitation (co-IP) and western blotting

Cells were washed twice with cold phosphate-buffered saline (PBS) and incubated in the lysis buffer for 1 h at 4 °C. The cell extracts were obtained by centrifuging at 12,000 × *g* for 10 min. Then, the protein samples were incubated with 2 μg of corresponding antibodies or normal rabbit/mouse IgG at 4 °C overnight with constant mixing. The protein A/G Sepharose beads were added to the protein samples and incubated for 4 h at 4 °C. Next, the beads were washed five times with cell lysis buffer. The immune complexes were subjected to SDS-PAGE, followed by immunoblotting with secondary antibodies. Immunodetection was performed using enhanced chemiluminescence (ECL System, Thermo Scientific) according to the manufacturer’s instructions. Full length original western blots for these results were provided in Supplementary File 1.

### Glutathione S-transferase (GST) pull-down experiments

GST-fused constructs were produced in BL21 *Escherichia coli*, and the bacterial lysates were collected using ultrasound. In vitro transcription and translation experiments were performed using rabbit reticulocytes (TNT system, Promega, USA) according to the manufacturer’s recommendations. In the GST pull-down assay, ~5 μg of the appropriate GST-fused protein with 25 μL glutathione-Sepharose beads were mixed with 40 μL of the in vitro transcribed/translated products and the protease inhibitor cocktail by constant rotation at 4 °C for 2 h. Beads were washed five times with binding buffer and then resuspended in 25 μL of 2 × SDS-PAGE loading buffer. The fused protein was measured by western blotting.

### RNA-sequencing analysis

MDA-MB-231 cells were transfected with siRNA RUNX2 for 48 h. Total RNA was extracted using TRIzol reagent (Roche, Switzerland) according to the manufacturer’s instructions. The product was sent to the Beijing Genomics Institute (BGI, Beijing, China) for mRNA library construction and sequencing. The sequencing data were verified by a quality control step and analyzed using R packages, including the DESeq2, clusterProfiler, and ggplot2 packages. Genes with a fold change of 1.5 and *p* < 0.05 were identified as differential genes, and raw data is available on GSE190249.

### Real-time quantitative PCR (RT-qPCR) analysis

Total cellular RNA was extracted from MDA-MB-231 cells using TRIzol reagent (Roche) according to the manufacturer’s instructions. cDNA was obtained by reverse transcription using the RevertAid First Strand cDNA Synthesis Kit (Roche). Relative quantitation was performed using the ABI PRISM 7500 System (Applied Biosystems, Foster City, CA, USA) by measuring the fluorescence of real-time SYBR green. Quantitation was performed using the comparative *C*_t_ method (2^−ΔΔCt^) with the expression of *Actin* as an internal control. The primers used were listed in Supplementary Table S3.

### ChIP-sequencing (ChIP-seq) analysis and quantitative ChIP (qChIP)

ChIP-seq analysis was performed using cleavage under targets and tagmentation (CUT&Tag, NOVOPROTEIN, Shanghai, China). All procedures strictly followed the kit instructions, as described previously [27]. Briefly, 1 × 10^6^ MDA-MB-231 cells were washed and bound with concanavalin A-coated magnetic beads (Bangs Laboratories, IN, USA). The bead-bound cells were resuspended in 50–100 µL dig-wash buffer and incubated with 4 μg anti-MTA1 and anti-RUNX2 primary antibodies. The primary antibody was removed, followed by incubation with the secondary antibody. Next, cells were resuspended in Tagmentation buffer and incubated at 37 °C for 1 h. Ampure XP beads were added to each tube by vortexing, and quickly spun to extract the DNA. Beads were washed and eluted using 30–40 µL of 10 mM Tris at pH 8. The elution liquid was used for library construction and high-throughput sequencing. For the qChIP assay, 1 × 10^7^ MDA-MB-231 cells were cross-linked with 1% formaldehyde, sonicated, pre-cleared, and incubated with 4 μg of antibody per reaction. Complexes were washed with low-and high-salt concentration buffers, and the DNA was extracted for qChIP assay using the QIAquick PCR Purification Kit. The specific primers used for the qPCR assay were supplied in Supplementary Table S4. ChIP-seq results are available on GSE190248.

### Colony formation assay

Cells were treated as indicated. A total of 8000 cells were maintained in culture media in 6-well dishes for approximately 14 days and then stained with crystal violet.

### 5-ethynyl-20-deoxyuridine (EdU) assay

Cells with respective treatments were seeded in 96-well plates and subjected to the EdU assay (C10310, RiboBio Co., Guangzhou, China) to detect proliferation using fluorescence detection. Before fixation, the cells were incubated in a conditioned medium from the kit, and the EdU assay was performed according to the manufacturer’s instructions.

### Chemotactic migration assay

Transwell inserts (BD Biosciences) were used to mimic the bone microenvironment to estimate the chemotactic migration of breast cancer cells. MC3T3-E1 cells were pre-seeded in the lower chamber of the 24-well plates. MDA-MB-231 or SUM159 cells (2 × 10^4^) were seeded in the upper chamber. The cells were co-cultured for 24 h. Then, migrating cells on the lower surface of the membrane in the upper chamber were fixed, washed, and stained with crystal violet. Images of invasive cells were captured using a light microscope. Three high-powered fields were counted for each membrane.

### Cancer cell–bone matrix adhesion assay

MC3T3-E1 cells were pre-seeded in 24-well plates with the culture medium containing 10% FBS for 10 days to produce bone matrix. 2 × 10^5^ MDA-MB-231 or SUM159 cells pre-labeled with GFP were added to the MC3T3-E1 cells layer and incubated for 5 min. Floating cells were aspirated. The adhesion efficiency was determined by dividing the number of adherent cells by the initial number of cancer cells.

### Osteoclastogenesis assay

RAW264.7 cells (2 × 10^4^) were plated in a 6-well culture plate and supplemented with 50 ng/mL receptor activator of nuclear factor-κ B ligand (RANKL, R&D System, USA) for 3 days. In the co-culture experiment, a conditional medium (CM) with RANKL from MDA-MB-231 or SUM159 cells was added, and the cells were co-cultured for an additional 7 days. Finally, the cells were harvested. Total RNA was extracted from cells. Osteoclastogenesis-related genes were analyzed by RT-qPCR.

### Spheroid-forming assays

A total of 5000 cells were plated in six-well ultralow attachment plates in serum-free DMEM-F12 supplemented with 0.4% BSA, B27 (50×, Invitrogen), 20 ng/mL bFGF, 10 ng/mL EGF, and 5 µg/mL insulin (Invitrogen). Fresh aliquots of stem cell medium were added every three days. Mammospheres were observed on day 5, which then increased in size and cell number until day 15.

### Mouse xenograft models

For the tumor initiation study, MDA-MB-231 cells were infected with lentivirus carrying a control shRNA or shRUNX2. These cells were injected with Matrigel (BD Biosciences) into the #4 mammary fat pads of 6-week-old female NOD/SCID mice at limiting dilutions of 500 and 100 cells. Five mice were assayed per group. Tumor growth was monitored for 2.5 months. For the primary tumorigenesis study, 6-week-old female NOD/SCID mice were randomly assigned to four groups (*n* = 3), and cells were infected with Vector and shSCR, RUNX2 and shSCR, RUNX2 and shMTA1, and RUNX2 and shCUL4B. A total of 5 × 10^6^ cells of each type were inoculated into the left abdominal mammary fat of mice. Sample sizes were based in standard protocols in the field. Experiments were blinded to the person performing measurement. Animal handling and procedures were approved by the Institutional Animal Care Center of the Capital Medical University.

### Tissue specimens and immunohistochemistry

Six patients diagnosed with breast cancer were recruited for this study. All human tissues were collected using protocols approved by the Ethics Committee of Cancer Hospital of Chinese Academy of Medical Sciences and Peking Union Medical College Cancer, and informed consent was obtained from all patients. The clinical characteristics of the patients were presented in Supplementary Table S5. Samples were frozen in liquid nitrogen immediately after surgical removal. Samples were fixed in 4% paraformaldehyde (Sigma–Aldrich) at 4 °C overnight and then processed, paraffin-embedded, sectioned 8-μm-thick sample sections, and stained with corresponding antibodies according to a standard protocol. Staining was completed via incubation with diaminobenzidine (DAB) substrate for 5–10 min, and monitored microscopically.

### Statistical analysis

All the results were based on at least three replicates. Cell results were presented as the mean ± SD and analyzed using GraphPad Prism (version 8.0, Graph Pad Software Inc., USA). Animal and clinical results were presented as mean ± SEM. Student’s *t* test was used to compare the difference between two groups, one-way ANOVA was used to compare the significance among three or more groups. Underlying assumptions for these tests, including sample independence, variance equality, and normality were assumed to be met although not explicitly examined. The level of significance was set at 5% for all tests.

## Results

### Upregulation of RUNX2 is correlated with breast cancer progression

**Fig. 1:**
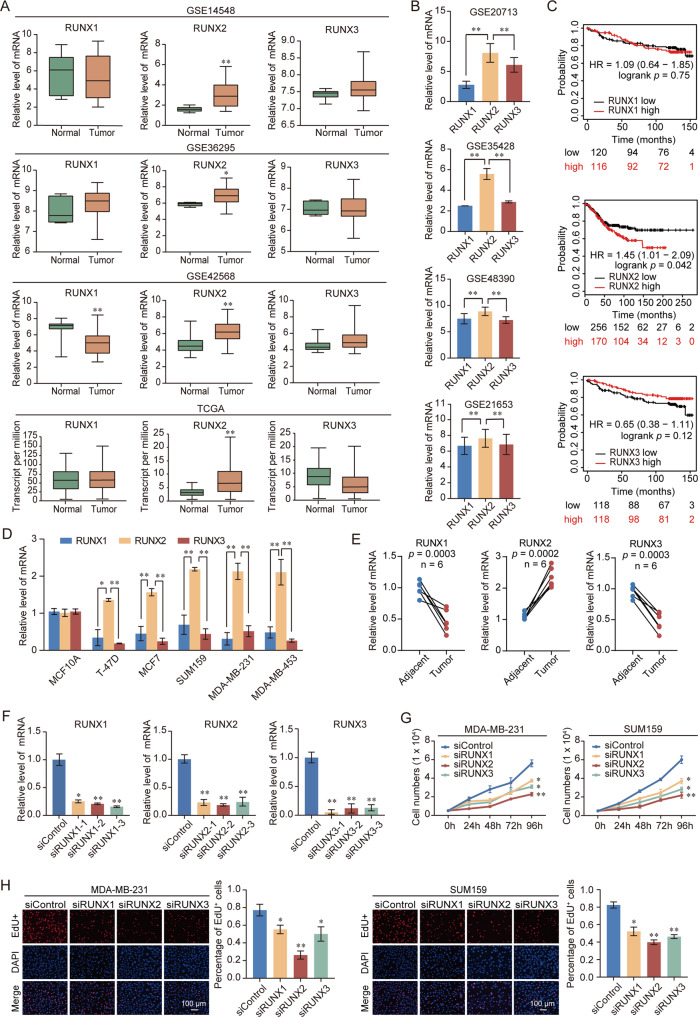
Upregulation of RUNX2 is correlated with breast cancer progression. **A** Analysis of RUNX family expression in normal and breast cancer by using GEO and TCGA database. **B** Analysis of RUNX family expression in breast cancer by using GEO database. **C** Kaplan–Meier survival analysis of the relationship between survival time and expression of RUNX family using an online tool (http://kmplot.com/analysis/). **D** Expression of RUNX family in normal and breast cancer cell lines. **E** Expression of RUNX family in breast cancer and adjacent tissues (*n* = 6). Results were presented as mean ± SEM. **F** Knockdown efficiencies of RUNX1, RUNX2, and RUNX3 were verified by RT-qPCR. **G** Growth curve assays were performed in MDA-MB-231 and SUM159 cells transfected with siRNA against RUNX1, RUNX2, and RUNX3. **H** EdU assays were performed in RUNX family-deficient MDA-MB-231 and SUM159 cells and corresponding statistical analysis. Representative images in each group were shown. Results were presented as mean ± SD. Two-tailed unpaired *t* test, *p < 0.05, **p < 0.01.

To determine the critical role of RUNX family members in the regulation of breast cancer progression, the expression status of each member of the RUNX family in breast cancer was evaluated using Gene Expression Omnibus (GEO) and The Cancer Genome Atlas (TCGA) database. These analyses indicated that only RUNX2 expression was significantly increased in breast cancer compared to normal samples (Fig. 1A). In breast cancer, the expression of RUNX2 was higher than that of RUNX1 and RUNX3 (Fig. 1B). Patients with lower expression of RUNX2 had a longer overall survival (OS) (Fig. 1C). Consistent with the bioinformatics analysis, the expression of RUNX2 was highest in the RUNX family in clinical breast cancer samples and cell lines; Moreover, RUNX2 was overexpressed, while RUNX1 and RUNX3 were decreased in breast cancer tissues (Fig. 1D, E). Depletion of RUNX2 had a stronger inhibitory effect on the proliferation of breast cancer cells than the suppression of RUNX1 or RUNX3 (Fig. 1F–H). These results suggested that RUNX2 might play a prominent role in breast cancer progression.

### RUNX2 regulates tumor suppressor genes expression and participates in bone metastasis related signaling pathways

To determine how RUNX2 regulated breast cancer cell growth, we performed RNA sequencing (RNA-seq) experiments in MDA-MB-231 cells using siRNA against RUNX2. A total of 1 947 downregulated genes and 2 564 upregulated genes were identified (fold change > 1.5; *p* < 0.05) in RUNX2-depleted cells (Supplementary Fig. 1A–C). Kyoto Encyclopedia of Genes and Genomes (KEGG) pathway analysis of the differential genes revealed that downregulated and upregulated genes were involved in vital biological processes, including HIF-1 signaling pathway, PI3K-AKT signaling pathway, osteoclast differentiation, IL-17 signaling pathway, and apoptosis (Supplementary Fig. 1D). Gene set enrichment analysis (GSEA) showed that the differentially expressed genes were enriched in TGF-β signaling pathway, PI3K-AKT-mTOR signaling pathway, fatty acid metabolism, and apoptosis (Supplementary Fig. 1E). We found that several well-known tumor suppressor genes were upregulated after the depletion of RUNX2, including *BAX*, *EIF3F*, *FADD*, *PPAR*α, *FBXW7*, *HSP90B1*, *CASP 7*, *SIAH2*, *TNFAIP3*, *TSC22D1*, and *SOD2* (Supplementary Fig. 1F, G). In addition, several oncogenes were also downregulated in RUNX2-depleted cells (Supplementary Fig. 1F, G). Together, these data revealed that RUNX2 might be involved in breast cancer progression and bone metastasis.

### RUNX2 is physically associated with the NuRD(MTA1) and CRL4B complexes

**Fig. 2:**
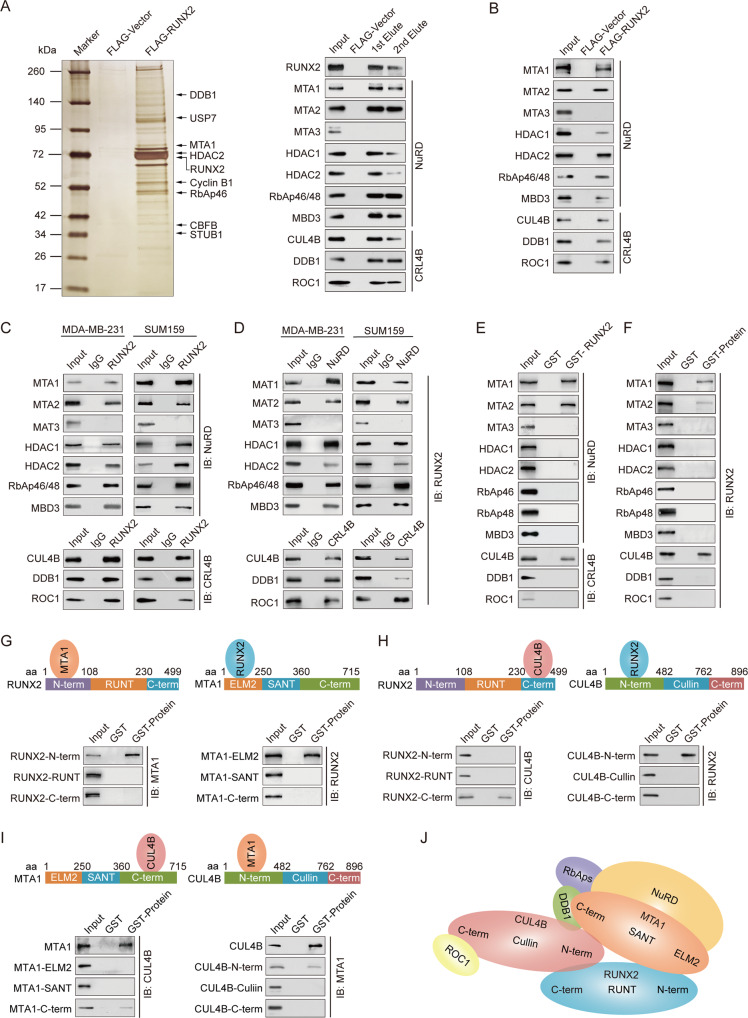
RUNX2 is physically associated with the NuRD(MTA1) complex and the CRL4B complex. **A** Immunoaffinity purification and mass spectrometry analysis of RUNX2-containing protein complexes. Whole-cell extracts from MDA-MB-231 cells stably expressing FLAG-Vector or FLAG-RUNX2 were immunopurified using anti-FLAG affinity columns and eluents with FLAG peptide. Elutes were resolved using SDS-PAGE and silver-stained. Protein bands were retrieved and analyzed using mass spectrometry. **B** Western blotting analysis of the purified fractions using antibodies against FLAG in MDA-MB-231 cells. **C** Immunoprecipitation with antibody against RUNX2 followed by immunoblotting with antibodies against MTA1, MTA2, MTA3, HDAC1, HDAC2, RbAp46/48, MBD3, CUL4B, DDB1, and ROC1. **D** Immunoprecipitation with antibodies against MTA1, MTA2, MTA3, HDAC1, HDAC2, RbAp46/48, MBD3, CUL4B, DDB1, and ROC1 followed by immunoblotting with antibody against RUNX2. **E**, **F** GST pull-down assays with GST-fused proteins expressed in bacteria and in vitro translated proteins as indicated. **G**–**I** Identification of the essential domains of RUNX2, MTA1, or CUL4B required for interaction. **J** Schematic diagram depicting molecular interactions among RUNX2/NuRD(MTA1)/CRL4B complex. IB, immunoblotting; aa, amino acid.

To better understand the molecular mechanism of RUNX2 in breast cancer, affinity purification and mass spectrometry analysis were performed to identify potential co-functional proteins. The results indicated that RUNX2 was co-purified with MTA1, HDAC2, RbAp46, DDB1, CBFB1, USP7, Cyclin B1, and STUB (Fig. 2A, left). Some of these proteins were reported previously, such as CBFB1, USP7, Cyclin B1, and STUB1 [28–31]. The proteins of NuRD and CRL4B complexes were confirmed by western blotting analysis (Fig. 2A, right). We further performed co-IP experiments to confirm the RUNX2-associated complexes. The results showed obvious interactions between RUNX2, the NuRD(MTA1) complex and the CRL4B complex (Fig. 2B). Interestingly, RUNX2 did not interact with MTA3. These results revealed that RUNX2 interacted with the MTA1-associated NuRD and CRL4B complexes (Fig. 2C, D).

To further investigate the physical association between RUNX2, NuRD, and CRL4B complexes, GST pull-down experiments were performed by incubating GST-fused RUNX2 with in vitro transcribed/translated MTA1, MTA2, MTA3, HDAC1, HDAC2, RbAp46, RbAp48, MBD3, CUL4B, DDB1, and ROC1. The results showed that RUNX2 interacted directly with MTA1, MTA2, and CUL4B but not with other components of the NuRD and CRL4B complexes (Fig. 2E, F). Next, to confirm the conjunct domain of RUNX2/MTA1/CUL4B complex, GST pull-down assays with GST-fused RUNX2 N-terminal fragment (1–108 amino acid [aa], RUNX2-N-term), the RUNT domain (108–230 aa, RUNX2-RUNT), and C-terminal fragment (230–499 aa, RUNX2-C-term), and in vitro transcribed/translated MAT1 or CUL4B demonstrated that the RUNX2-N-term and RUNX2-C-term were responsible for its interaction with MTA1 and CUL4B, reciprocally (Fig. 2G, H). GST-fused MTA1 ELM2 (1–250 aa, MTA1-ELM2), SANT domain (250–360 aa, MTA1-SANT), C-terminal fragment (360–715 aa, MTA1-C-term), and in vitro transcribed/translated RUNX2 or CUL4B demonstrated that the MTA1-ELM2 and MTA1-C-term were responsible for its interaction with RUNX2 and CUL4B, respectively (Fig. 2G, I). Moreover, GST-fused CUL4B N-terminal fragment (1–482 aa, CUL4B-N-term), cullin domain (482–762 aa, CUL4B-cullin), C-terminal fragment (482–896 aa, CUL4B-C-term), and in vitro transcribed/translated RUNX2 or MTA1 demonstrated that CUL4B-N-term was responsible for its interaction with RUNX2 and MTA1 (Fig. 2H, I). Together, these results not only provided further support for the specific interaction among RUNX2, the NuRD(MTA1) complex and the CRL4B complexes but also delineated the molecular details involved in the formation of the RUNX2/NuRD(MTA1)/CRL4B complex, as schematically summarized (Fig. 2J). The detailed results of the mass spectrometric analysis are shown in Supplementary Table S6.

### The RUNX2/NuRD(MTA1)/CRL4B complex promotes proliferation and drives attraction and adhesion of breast cancer cells to bone

**Fig. 3:**
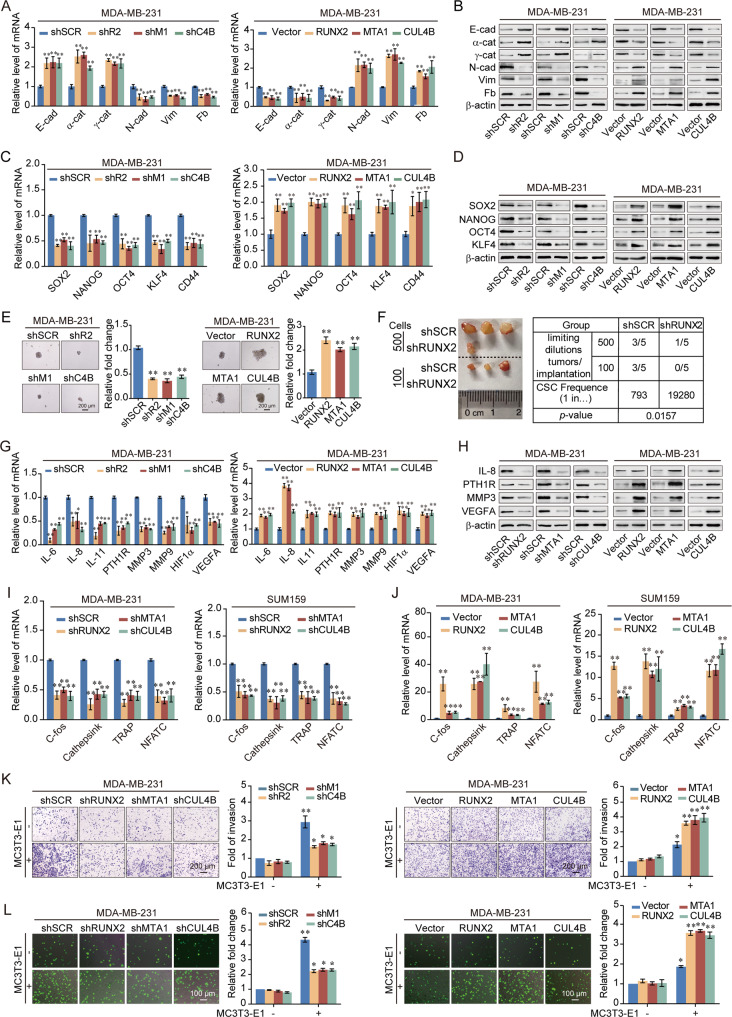
RUNX2 recruits the NuRD(MTA1)/CRL4B complex to promote the epithelial-to-mesenchymal transition, stemness, and bone metastasis of breast cancer cells. **A** RT-qPCR results for the relative mRNA expression of epithelial and mesenchymal markers in MDA-MB-231 cells with depletion or overexpression of RUNX2, MTA1, or CUL4B. **B** Western blotting analysis of epithelial and mesenchymal markers in MDA-MB-231 cells with depletion or overexpression of RUNX2, MTA1, or CUL4B. **C** RT-qPCR results for the relative mRNA expression of stem cell markers in MDA-MB-231 cells with depletion or overexpression of RUNX2, MTA1, or CUL4B. **D** Western blotting analysis of stem cell markers in MDA-MB-231 cells with depletion or overexpression of RUNX2, MTA1, or CUL4B. **E** MDA-MB-231 cells stably knocked down or overexpressed RUNX2, MTA1, or CUL4B. Representative images of spheres were grown in suspension culture for 15 days. Cells were placed in an ultra-low attachment six-well plate (5000/well). **F** Tumorigenicity was tested by injecting MDA-MB-231-shSCR or MDA-MB-231-shRUNX2 cells into the mammary gland fat pads of NOD/SCID mice at various dilutions (*n* = 5 in each group). The stem cell frequency in xenograft tumors was calculated using the Extreme Limiting Dilution Analysis (ELDA) software (http://bioinf.wehi.edu.au/software/elda/index.html), results were presented as mean ± SEM. **G** RT-qPCR results for the relative mRNA expression of bone metastasis-related markers in MDA-MB-231 cells with depletion or overexpression of RUNX2, MTA1, or CUL4B. **H** Western blotting analysis of bone metastasis-related markers in MDA-MB-231 cells with depletion or overexpression of RUNX2, MTA1, or CUL4B. **I**, **J** RT-qPCR results for the relative mRNA expression of osteoclastogenesis markers in RAW264.7 cells co-cultured with the conditional medium of MDA-MB-231 and SUM159 cells with depletion or overexpression of RUNX2, MTA1, or CUL4B. **K**, **L** Chemotactic migration assays (**K**) and cancer cell–bone matrix adhesion assays (**L**) of MDA-MB-231 cells with depletion or overexpression of RUNX2, MTA1, or CUL4B co-cultured with MC3T3-E1 cells. Representative images in each group were shown. shR2, shRUNX2; shM1, shMTA1; shC4B, shCUL4B; E-cad, E-cadherin; *α*-cat, *α*-catenin; *γ*-cat, *γ*-catenin; N-cad, N-cadherin; Vim, Vimentin; Fb, Fibronectin. All results were presented as mean ± SD. Two-tailed unpaired *t* tes*t*, *p < 0.05, **p < 0.01.

In order to further elucidate the physical and functional interactions among RUNX2, NuRD(MTA1), and CRL4B, we investigated the role of the RUNX2/NuRD(MTA1)/CRL4B complex in terms of EMT, stemness, and bone metastasis of breast cancer. We found that RUNX2, MTA1, or CUL4B depletion inhibited the proliferation of cancer cells, whereas RUNX2, MTA1, or CUL4B overexpression significantly promoted proliferation (Supplementary Fig. 2A–H). To further investigate the role of RUNX2, MTA1, or CUL4B in EMT, the expression of EMT-related markers was analyzed. RUNX2, MTA1, or CUL4B depletion led to a reduction in mesenchymal markers fibronectin, N-cadherin, and vimentin and an induction in epithelial markers E-cadherin, α-catenin, and γ-catenin, whereas RUNX2, MTA1, or CUL4B overexpression led to the opposite result (Fig. 3A, B, Supplementary Fig. 3A, B). Previous studies showed that EMT was involved in the generation and maintenance of stem-like cell properties [32, 33]. We have demonstrated that CUL4B could upregulate the breast cancer stem cell population [25]. Thus, we investigated whether RUNX2 or MTA1 affect stem-like phenotypes in breast cancer cells. The stem cell markers were downregulated with RUNX2, MTA1, or CUL4B knockdown and upregulated while stably expressing RUNX2, MTA1, or CUL4B (Fig. 3C, D, Supplementary Fig. 3C, D). Spheroid-forming assays also indicated that RUNX2, MTA1, or CUL4B knockdown suppressed the volume of spheres, while RUNX2, MTA1, or CUL4B overexpression promoted them (Fig. 3E, Supplementary Fig. 3E). Cells stably expressing shRUNX2 had markedly inhibited tumor-initiating capacity (Fig. 3F, Supplementary Fig. 3F). Previous RNA-seq analysis of RUNX2 indicated that differential genes were involved in bone metastasis related pathways. Bone metastasis-related markers expression was decreased by the knockdown of RUNX2, MTA1, or CUL4B but increased with the overexpression of RUNX2, MTA1, or CUL4B (Fig. 3G, H, Supplementary Fig. 3G, H). Furthermore, the expression of osteoclastogenesis-related genes showed similar results (Fig. 3I, J). The chemotactic migration assay and cancer cell–bone matrix adhesion assay indicated that depletion of RUNX2, MTA1, or CUL4B hampered breast cancer cell metastasis to the bone, while overexpression of RUNX2, MTA1, or CUL4B resulted in the opposite trend (Fig. 3K, L, Supplementary Fig. 3I, J). These results suggested that RUNX2, MTA1, or CUL4B could promote the proliferation, EMT, stemness, and bone metastasis potential of breast cancer cells.

**Fig. 4:**
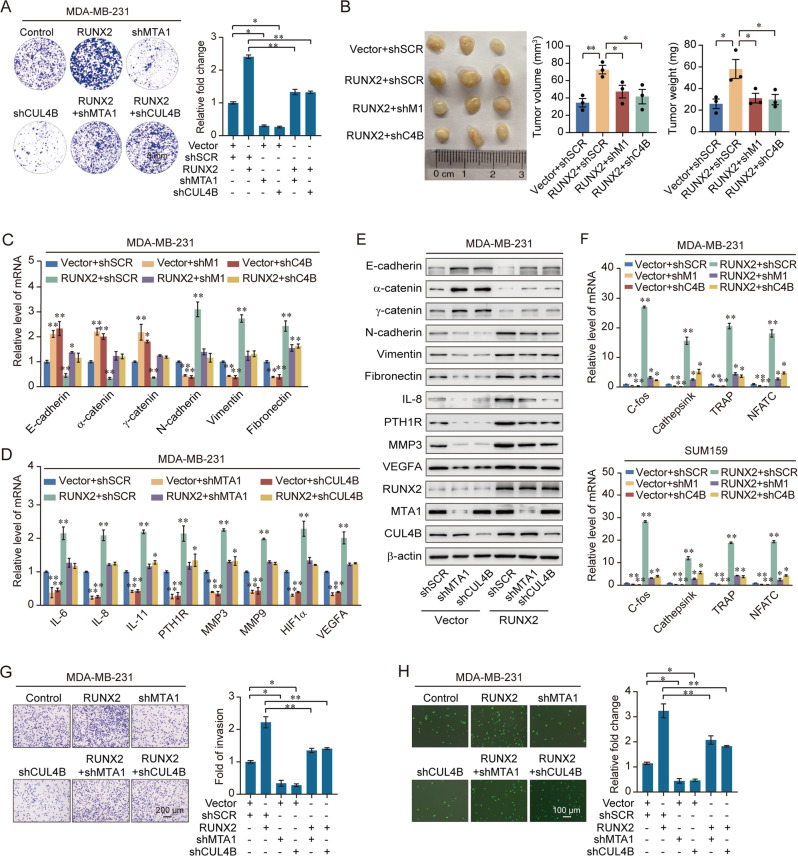
The RUNX2/NuRD(MTA1)/CRL4B complex promotes proliferation and drives the attraction and adhesion of breast cancer cells to bone. **A** Colony formation assays were performed in MDA-MB-231 cells with overexpression of RUNX2 and co-transfected with shMTA1 or shCUL4B. **B** MDA-MB-231 cells infected with lentiviruses carrying the indicated expression constructs and/or shRNAs were inoculated orthotopically into the abdominal mammary fat pad of 6-week-old female NOD/SCID mice (*n* = 3); the tumor volume: length × width²; Results were presented as mean ± SEM. **C** RT-qPCR results for the relative mRNA expression of epithelial and mesenchymal markers in MDA-MB-231 cells with overexpression of RUNX2 and co-transfected with shMTA1 or shCUL4B. **D** RT-qPCR results for the relative mRNA expression of bone metastasis-related markers in MDA-MB-231 cells with overexpression of RUNX2 and co-transfected with shMTA1 or shCUL4B. **E** Western blotting analysis of epithelial and mesenchymal and bone metastasis-related markers in MDA-MB-231 cells with overexpression of RUNX2 and co-transfected with shMTA1 or shCUL4B. **F** RT-qPCR results for the relative mRNA expression of osteoclastogenesis markers in RAW264.7 cells co-cultured with the conditional medium of MDA-MB-231 and SUM159 cells with overexpression of RUNX2 and depletion of MTA1 or CUL4B. **G**, **H** Chemotactic migration assays (**G**) and cancer cell–bone matrix adhesion assays (**H**) of MDA-MB-231 cells. These cells were co-cultured with MC3T3-E1 cells after knockdown of MTA1 or CUL4B, and overexpression of RUNX2 and co-transfected with shMTA1 or shCUL4B. shM1, shMTA1; shC4B, shCUL4B. Representative images in each group were shown. Results were presented as mean ± SD. Two-tailed unpaired *t* test, *p < 0.05, **p < 0.01.

The colony formation assays indicated that increased proliferation induced by overexpressing RUNX2 was decreased by simultaneously knocking-down MTA1 or CUL4B (Fig. 4A, Supplementary Fig. 4A). More importantly, to investigate the role of the RUNX2/NuRD(MTA1)/CRL4B complex in breast cancer in vivo, the primary tumorigenesis results showed that the volume of the primary tumor was significantly increased with the overexpression of RUNX2 but was inhibited by the simultaneous knockdown of MTA1 or CUL4B (Fig. 4B, Supplementary Fig. 4B). We also found that depletion of either MTA1 or CUL4B resulted in induction of epithelial markers and reduction of mesenchymal markers and bone metastasis genes, and depletion of either MTA1 or CUL4B combined with overexpression of RUNX2 restored the expression of these markers (Fig. 4C–E, Supplementary Fig. 4C–E). Consistently, the expression of osteoclastogenesis-related genes showed similar results (Fig. 4F). Morphologically, MTA1 or CUL4B depletion reduced bone metastasis by RUNX2 (Fig. 4G, H, Supplementary Fig. 4F, G). Thus, these results supported the critical roles of the RUNX2/NuRD(MTA1)/CRL4B complex in modulating EMT and bone metastasis in breast cancer.

### RUNX2 recruits the NuRD(MTA1)/CRL4B complex for transcriptional repression in breast cancer cells

**Fig. 5:**
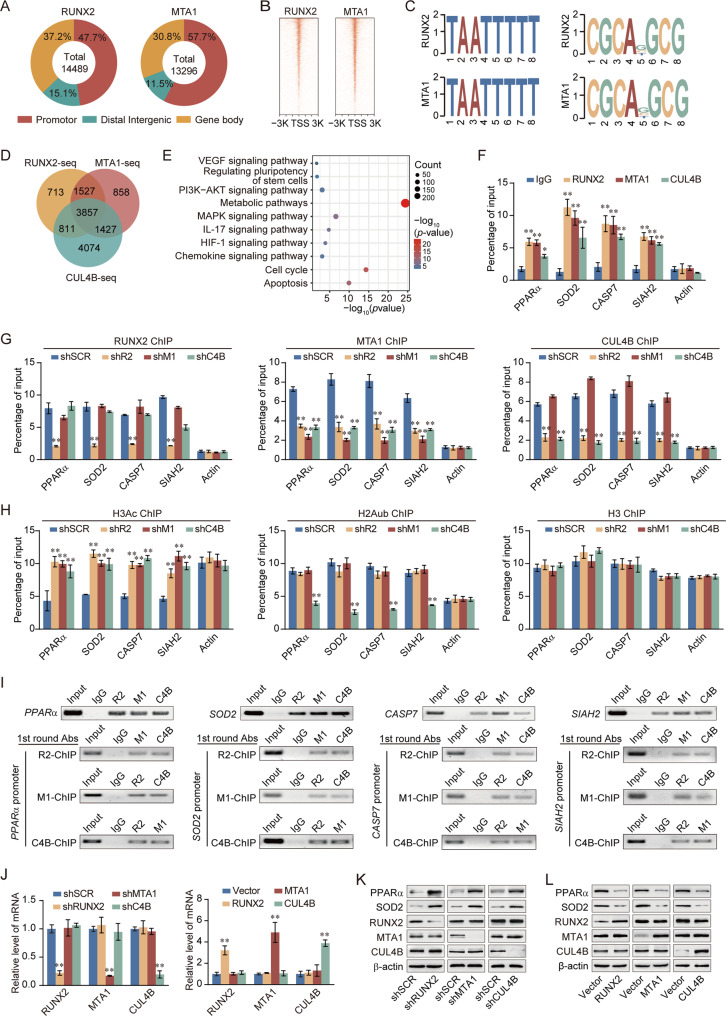
RUNX2 recruits the NuRD(MTA1)/CRL4B complex for transcriptional repression in breast cancer cells. **A** Genomic distribution of RUNX2 and MTA1 determined using ChIP-seq analysis. **B** ChIP-seq density heatmaps of RUNX2 and MTA1. **C** RUNX2 and MTA1-bound motifs analyzed using the MEME suite. **D** Venn diagram of overlapping promoters bound by RUNX2/MTA1/CUL4B complex. **E** KEGG pathways analysis of 3857 unique genes. **F** Verification of ChIP-seq results using qChIP analysis of indicated genes. **G**, **H** MDA-MB-231 cells were infected with lentiviruses carrying the indicated shRNAs. qChIP analysis of the target gene promoters was performed using antibodies against RUNX2, MTA1, or CUL4B (**G**) or against histone H3Ac and H2AK119ub1 (**H**); Histone H3 was detected as an internal control. Results were represented as fold change over control with β-actin as a negative control. **I** ChIP and Re-ChIP experiments in MDA-MB-231 cells with the indicated antibodies. **J** Knockdown and overexpression efficiencies of RUNX2, MTA1, or CUL4B verified by RT-qPCR. **K**, **L** Western blotting analysis of ChIP-seq indicated genes (PPARα and SOD2) in MDA-MB-231 cells with depletion or overexpression of RUNX2, MTA1, or CUL4B. TSS, transcriptional start site; shR2, shRUNX2; shM1, shMTA1; shC4B, shCUL4B; R2, RUNX2; M1, MTA1; C4B, CUL4B; H3Ac, pan-H3 acetylation; H2Aub, H2AK119 monoubiquitination; 1st round Abs, first round antibodies. All results were presented as mean ± SD. Two-tailed unpaired *t* test, *p < 0.05, **p < 0.01.

To better understand the biological significance of the RUNX2/NuRD(MTA1)/CRL4B complex, we analyzed the occupancy of genome-wide transcriptional targets of RUNX2, MTA1. We found 10 455 RUNX2- and MTA1-specific binding peaks (Fig. 5A, B). Moreover, we found that RUNX2 and MTA1 had similar binding motifs (Fig. 5C), supporting the notion that they physically interacted and are functionally linked. Next, enriched genes from RUNX2 and MTA1 were analyzed for overlapping DNA promoter sequences with CUL4B [25]; these promoters represented co-targets of the RUNX2/NuRD(MTA1)/CRL4B complex (Fig. 5D). A total of 3857 unique genes were identified that were enriched in several KEGG pathways in accordance with RNA-seq analysis, including the PI3K-AKT signaling pathway, HIF-1 signaling pathway, and IL-17 signaling pathway (Fig. 5E). qChIP analysis showed that the RUNX2/NuRD(MTA1)/CRL4B complex strongly enriched the promoters of tumor suppressor genes (*SOD2*, *CASP7*, *PPAR*α, *BAX*, *SIAH2*, *ANXAN7*, *FBAW7*, *EIF3F*, *EI24*, *TSC22D1*, *EGR1*, and *NEURL1*) (Fig. 5F, Supplementary Fig. 5A). In addition, RT-qPCR analysis showed that RUNX2, MTA1, or CUL4B knockdown enhanced, while overexpression suppressed the expression of these tumor suppressor genes (Supplementary Fig. 5B, C).

To support the notion that the RUNX2, MTA1, and CUL4B occupied the target promoters in the context of the RUNX2/NuRD(MTA1)/CRL4B complex, qChIP experiments were performed and indicated that depletion of either RUNX2, MTA1, or CUL4B resulted in a marked reduction in the recruitment of the corresponding proteins at the promoters of the target genes (Fig. 5G, Supplementary Fig. 5D). Suppression of RUNX2 resulted in a significant reduction in the recruitment of MTA1 and CUL4B to the target gene promoters, whereas shMTA1 had little effect on the recruitment of RUNX2 and CUL4B at the target gene promoters (Fig. 5G, Supplementary Fig. 5D). Knockdown of CUL4B led to a dramatic decrease in the recruitment of MTA1, but not RUNX2, at the target gene promoters (Fig. 5G, Supplementary Fig. 5D). Knockdown of either RUNX2 or MTA1 consistently led to a dramatic increase in histone pan-H3 acetylation (H3Ac) at the promoters of target genes (Fig. 5H, Supplementary Fig. 5E); however, knockdown of either RUNX2 or MTA1 led to a limited reduction in the abundance of the histone H2K119 monoubiquitination (H2AK119ub1) with these promoters (Fig. 5H, Supplementary Fig. 5E), suggesting that CUL4B-mediated H2AK119ub1 acts in conjunction with MTA1-mediated H3Ac. These results confirmed that MTA1 and CUL4B were recruited to target gene promoters by RUNX2, supporting the existence of RUNX2, the NuRD(MTA1)/CRL4B complex in the same protein complex on target gene promoters and the functional coordination among these chromatin modifiers.

To further support the proposition that RUNX2 recruited the NuRD(MTA1)/CRL4B complex to form a protein complex at target promoters, sequential ChIP/Re-ChIP experiments were performed on four representative target genes PPARα, SOD2, CASP7, and SIAH2 (Fig. 5I). The results showed that the PPARα, SOD2, CASP7, and SIAH2 promoters that were immunoprecipitated with antibodies against RUNX2 could be re-immunoprecipitated with antibodies against MTA1 or CUL4B (Fig. 5I). Similar results were obtained when initial ChIP was performed with antibodies against MTA1 or CUL4B (Fig. 5I). In agreement with this, protein expression of PPARα and SOD2 increased with the deletion of RUNX2, MTA1, or CUL4B in MDA-MB-231 cells. On the contrary, the expression level decreased (Fig. 5J–L). Furthermore, the expression of PPARα and SOD2 was explored in normal and breast cancer tissues from published datasets (GSE42568, GSE14548, and GSE54002). As expected, both PPARα and SOD2 were significantly downregulated in breast cancer (Supplementary Fig. 6A). Kaplan–Meier survival analysis revealed that higher expression of PPARα and SOD2 was associated with improved survival of breast cancer patients (Supplementary Fig. 6B). These results confirmed that RUNX2, NuRD(MTA1), and CRL4B were functionally associated through transcriptional repression of a cohort of target genes (e.g., *PPAR*α, *SOD2*, *CASP7*, and *SIAH2)*.

### RUNX2 promotes the invasion and drives attraction and adhesion of breast cancer cells to the bone by inhibiting PPARα/SOD2 expressions

In order to gain further support for the notion that the RUNX2/NuRD(MTA1)/CRL4B complex promotes the EMT and bone metastasis of breast cancer cells through the transcriptional repression of target genes, indicated experiments were performed.

**Fig. 6:**
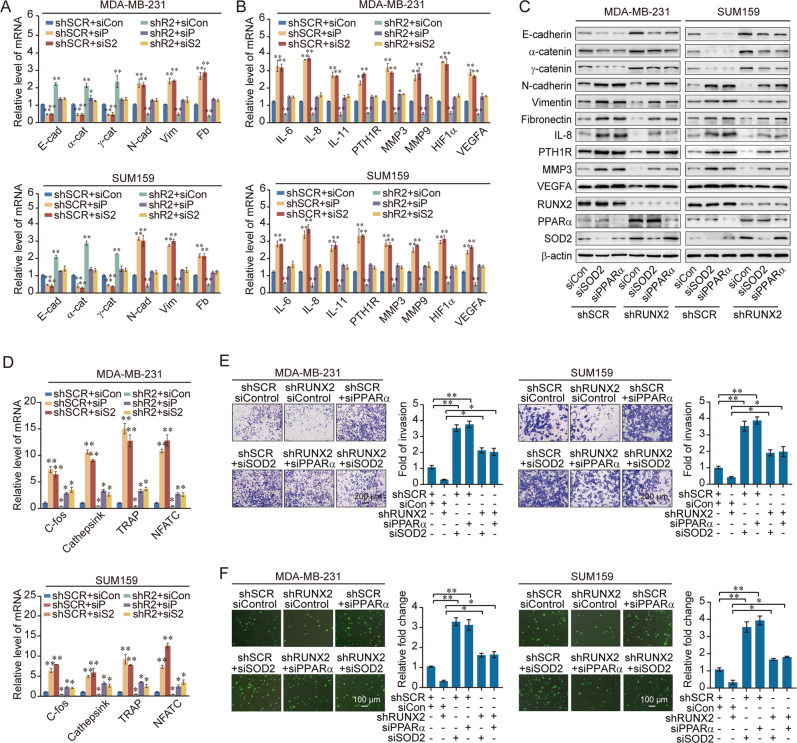
RUNX2 promotes the invasion and drives attraction and adhesion of breast cancer cells to the bone by inhibiting PPARα/SOD2 expressions. **A**–**C** Expression of the indicated epithelial and mesenchymal markers (**A**) and bone metastasis-related markers (**B**) were measured by RT-qPCR or western blotting (**C**) in MDA-MB-231 and SUM159 cells with depletion of RUNX2 and PPARα, or depletion of RUNX2 and SOD2. **D** RT-qPCR results for the relative mRNA expression of osteoclastogenesis markers in RAW264.7 cells co-cultured with the conditional medium of MDA-MB-231 and SUM159 cells with depletion of RUNX2 and PPARα, or depletion of RUNX2 and SOD2. **E**, **F** Chemotactic migration assays (**E**) and cancer cell–bone matrix adhesion assays (**F**) of MDA-MB-231 and SUM159 cells. These cells were co-cultured with MC3T3-E1 cells after depletion of RUNX2 and PPARα or SOD2. siCon, siControl; shR2, shRUNX2; siP, siPPARα; siS2, siSOD2; E-cad, E-cadherin; α-cat, α-catenin; γ-cat, γ-catenin; N-cad, N-cadherin; Vim, Vimentin; Fb, Fibronectin. Representative images in each group are shown. All results are presented as mean ± SD. Two-tailed unpaired *t* test, *p < 0.05, **p < 0.01.

We found that RUNX2 knockdown increased epithelial markers and decreased mesenchymal marker expression, which was partially rescued via the co-knockdown of PPARα, indicating that RUNX2 could promote breast cancer cells invasion by repressing PPARα expression (Fig. 6A, C, Supplementary Fig. 6C). Apparently, decreased bone metastasis-related markers could be rescued via the co-knockdown of PPARα (Fig. 6B, C). In addition, the suppressed osteoclastogenesis-related markers by shRUNX2 could be rescued through co-knockdown of PPARα (Fig. 6D). Similarly, experiments with RUNX2 depletion indicated that the inhibition of bone metastasis in breast cancer cells by knockdown of PPARα was dependent on RUNX2, at least partially (Fig. 6E, F). Similar results were found in knockdown of SOD2. These results suggested that RUNX2 promoted EMT and bone metastasis in breast cancer by inhibiting PPARα and SOD2 expression.

### The expression of RUNX2 is upregulated in multiple carcinomas and is the potential cancer biomarker

**Fig. 7:**
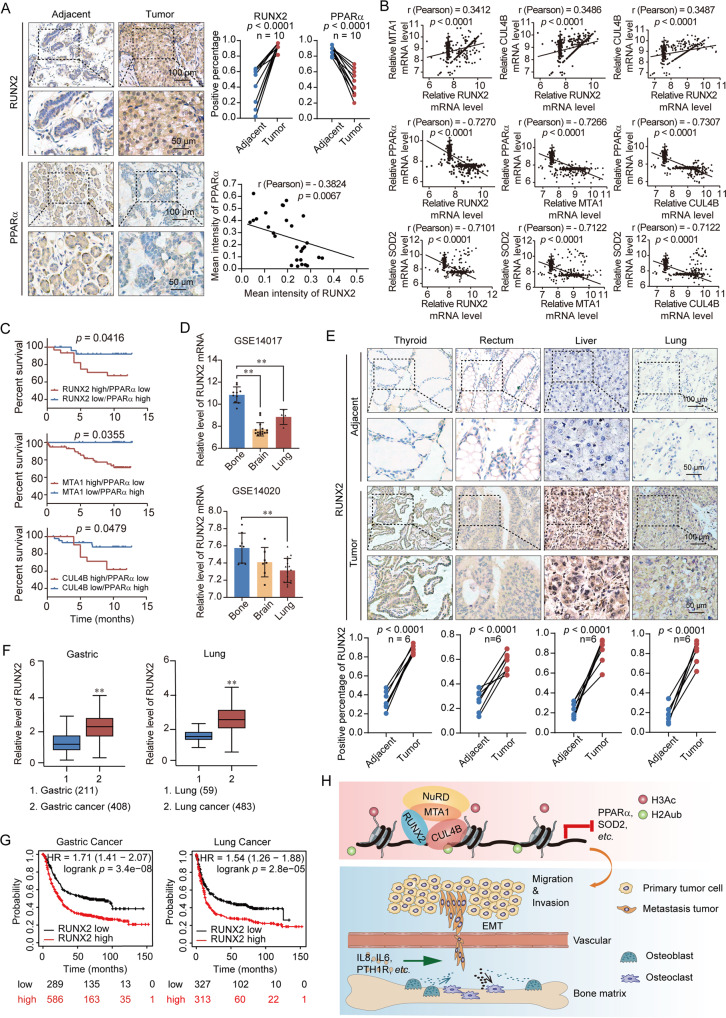
The expression of RUNX2 is upregulated in multiple carcinomas and is the potential cancer biomarker. **A** Immunohistochemical staining of RUNX2 and PPARα in breast cancer and adjacent tissues (*n* = 10). **B** Analysis of published clinical datasets (GSE48390) for the expression of RUNX2, MTA1, CUL4B, PPARα, and SOD2 by two-tailed unpaired *t* test. **C** Kaplan–Meier survival analysis of GSE3494 for the relationship between survival time and RUNX2/PPARα, MTA1/PPARα, and CUL4B/PPARα expression signatures in breast cancer. Survival curves were calculated using Kaplan–Meier method. Log-rank tests were used for the statistical analysis. **D** Analysis of a GEO dataset (GSE14017) for the expression of RUNX2 in breast cancer with bone, brain, or lung metastasis. **E** Immunohistochemical staining of RUNX2 in paired tumor tissues of thyroid, rectum, liver, and lung versus adjacent normal tissues (*n* = 6). **F** RUNX2 expression in gastric and lung cancers microarray datasets available from GAPIA (https://gepia.cancer-pku.cn/). **G** Kaplan–Meier survival analysis of the relationship between survival time and RUNX2 signature in gastric and lung cancers using the online tool (http://kmplot.com/analysis/). **H** The proposed regulatory mechanisms of the RUNX2/MTA1/CUL4B complex in controlling invasion and bone metastasis of breast cancer. All results were presented as mean ± SEM. Two-tailed unpaired *t* test, *p < 0.05, **p < 0.01.

Ten breast cancer samples were collected and performed tissue microarray analysis via immunohistochemical staining to examine the expression of RUNX2 and PPARα. We found that the expression of RUNX2 was concurrently upregulated in breast cancer samples. Consistent with our observation that PPARα was a downstream target of RUNX2, the expression of PPARα was found to be downregulated in these breast cancer samples and the level of its expression is negatively correlated with RUNX2 (Fig. 7A). The published breast cancer dataset (GSE48390) revealed a positive correlation between RUNX2, MTA1 and CUL4B, and negative correlations among RUNX2/MTA1/CUL4B and PPARα/SOD2 (Fig. 7B). Kaplan–Meier survival plot of RUNX2 and PPARα using GSE3494 showed patients with high expression of RUNX2, MTA1, or CUL4B while low expression of PPARα got a lower OS rate (Fig. 7C). The expression of RUNX2 was highest in breast cancer patients with bone metastasis (Fig. 7D). To investigate whether the effect of RUNX2 could be extended to a broader scope of cancers, we performed tissue microarray using immunohistochemical staining to analyze the expression of RUNX2 in 6 patients with other carcinomas (i.e., thyroid, rectum, liver, and lung)”. The results indicated that RUNX2 expression was significantly upregulated in these carcinomas (Fig. 7E). Furthermore, analysis of datasets from the GEPIA database (https://gepia.cancer-pku.cn/) revealed lower RUNX2 expression with improved survival compared to normal tissues in gastric and lung cancers (Fig. 7F, G). In summary, our results showed that RUNX2 expression was upregulated in multiple carcinomas including breast cancer, while PPARα was downregulated in breast cancer, suggesting the potential of RUNX2 as a biomarker of breast cancer.

## Discussion

In this study, We found that RUNX2 interacted with the NuRD(MTA1) complex and the CRL4B complex both physically and functionally. The ChIP assay indicated that the RUNX2/NuRD(MTA1)/CRL4B complex occupied a number of tumor suppressor gene promoters and was associated with increased histone deacetylation and ubiquitylation to function as a transcription inhibitor. Functional experiments demonstrated that RUNX2/NuRD(MTA1)/CRL4B complex could promote EMT and bone metastasis by inhibiting the expression of PPARα and SOD2 in breast cancer (Fig. 7H).

The increased expression of RUNX2 in breast cancer cells that metastasize to the bone indicates protumorigenic and pro-metastatic roles [34], although the detailed molecular mechanism is not very clear. RUNX2 can induce EMT and invasiveness of breast cancer cells partly by inhibiting SNAI2 expression [35, 36]. Notably, we showed that overexpression of RUNX2 not only promoted EMT but also stemness of breast cancer cells. Our previous studies demonstrated that the NuRD(MTA1)/CRL4B complex and the SIRT1/CRL4B complex promote stemness and EMT in breast cancer and pancreatic cancer, respectively [24, 25]. We have also demonstrated that NuRD(MTA1) complex physically associates with PRMT5 to promote the EMT process and strongly induced bone metastasis in cervical cancer [37]. Besides, RUNX2 also can activate Indian Hedgehog expression and further increase PTH1R levels in breast cancer metastatic bone disease [38]. In this regard, our current study also revealed the RUNX2/NuRD(MTA1)/CRL4B complex promoted PTH1R, IL-6, and IL-8 expression levels. Together, our study demonstrated that the RUNX2, NuRD(MTA1), and CRL4B complexes are physically associated and functionally linked to the promotion of EMT and bone metastasis of breast cancer cells. The genome-wide analysis demonstrated that the target genes of RUNX2/NuRD(MTA1)/CRL4B complex were enriched in multiple pathways related to bone metastasis, including the IL-17 signaling pathway [39]. IL-17 can help tumor cells escape from host immunosurveillance and promote metastasis by stimulating the release of multiple cytokines, such as IL-6, IL-8, and G-CSFs [40]. Therefore, it is suggested that RUNX2/NuRD(MTA1)/CRL4B complex might activate the IL-17 signaling pathway and multiple cytokines to induce bone metastasis in breast cancer.

The NuRD complex involved in the remodeling of nucleosomes and the deacetylation of histones, is known to suppress global gene expression [41]. In our previous studies, CUL4B worked by catalyzing H2AK119 monoubiquitination and coordinated with PRC2-catalyzed H3K27me3 and physically associated with four HDAC multiprotein complexes to be a transcriptional co-repressor [25, 42, 43]. According to the qChIP assays, knockdown of either RUNX2 or MTA1 consistently led to a dramatic increase in histone pan-H3 acetylation at the promoters of target tumor suppressor genes. Knockdown of CUL4B resulted in a noticeable decrease in H2AK119ub1 at the promoters of these genes. These results suggest that RUNX2/NuRD(MTA1)/CRL4B complex activates transcriptional repression involved in histone deacetylation and ubiquitylation and places RUNX2/MTA1/CUL4B at the node of the hierarchical regulatory network of EMT and bone metastasis.

PPARα plays a key role in energy homeostasis by modulating glucose and lipid metabolism, and tumorigenesis [44, 45]. Our results indicated that RUNX2/NuRD(MTA1)/CRL4B complex suppressed invasion, migration, and bone metastasis of breast cancer cells through inhibiting PPARα. Consistent with other studies, PPARα diverted energy away from Warburg-based tumor energy metabolism to lipogenesis to inhibit cell proliferation and tumor progression [46, 47]. Furthermore, PPARα agonists could lead to decreased vascularization, anti-inflammatory effects, and decreased levels of IL-1β and IL-6 [48, 49]. Combined with our results, RUNX2 could promote the expression of VEGFA, IL-6, and IL-8 though inhibited PPARα. Thus, RUNX2 and PPARα may be potential therapeutic targets for breast cancer bone metastasis.

In conclusion, we demonstrated that RUNX2 could cooperate with NuRD(MTA1)/CRL4B complex and acted as an inducer in various biological processes, including cell proliferation, invasion, bone metastasis, as well as cancer stemness of breast cancer. Furthermore, the RUNX2/NuRD(MTA1)/CRL4B complex contributed to the epigenetic silencing of tumor suppressors. PPARα and SOD2, which inhibited breast cancer bone metastasis, were found to be the target genes of the RUNX2/NuRD(MTA1)/CRL4B complex. Our research provides a new transcription regulatory model and a novel molecular basis for RUNX2 in carcinogenesis and metastasis, suggesting that RUNX2 is a potential therapeutic target for cancer treatment.

## Supplementary information

Supplementary material file Figures

Supplementary material file Tables

Supplementary material file Legends

Uncropped western blots related to results

Reproducibility Checklist

## Data Availability

Raw data for the RNA-seq (siRUNX2) and ChIP-seq results in this study are uploaded to Gene Expression Omnibus (GEO): GSE190249, GSE190248, respectively.
