# Supplementary material for: RUNX2 recruits the NuRD(MTA1)/CRL4B complex to promote breast cancer progression and bone metastasis
Source: Cell Death Differ. 2022 May 9;29(11):2203–17. doi: 10.1038/s41418-022-01010-2 (PMC9613664; doi:10.1038/s41418-022-01010-2)
Supplement: Supplementary file 1 — Supplementary material file Figures [file 41418_2022_1010_MOESM1_ESM.docx]

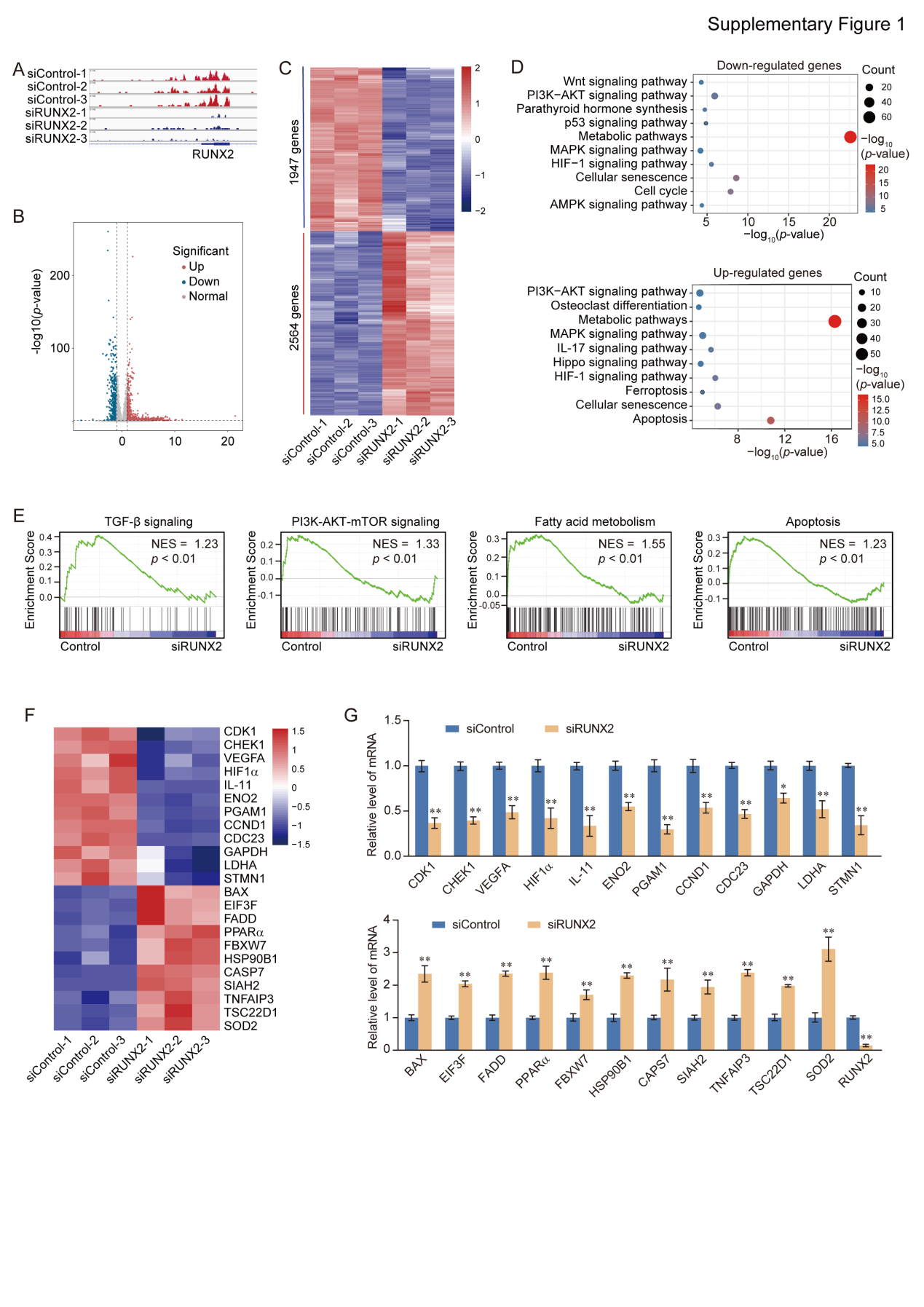


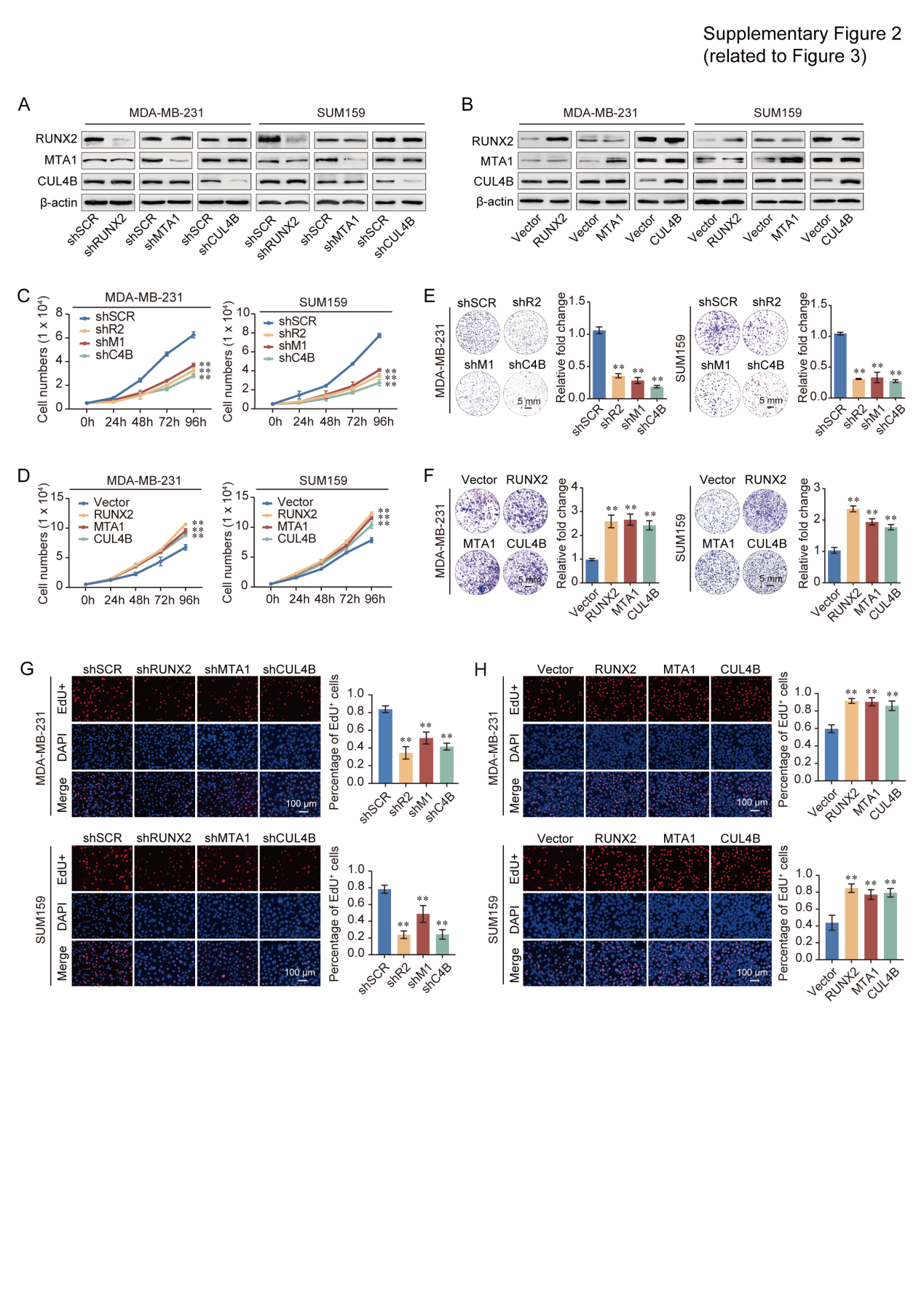


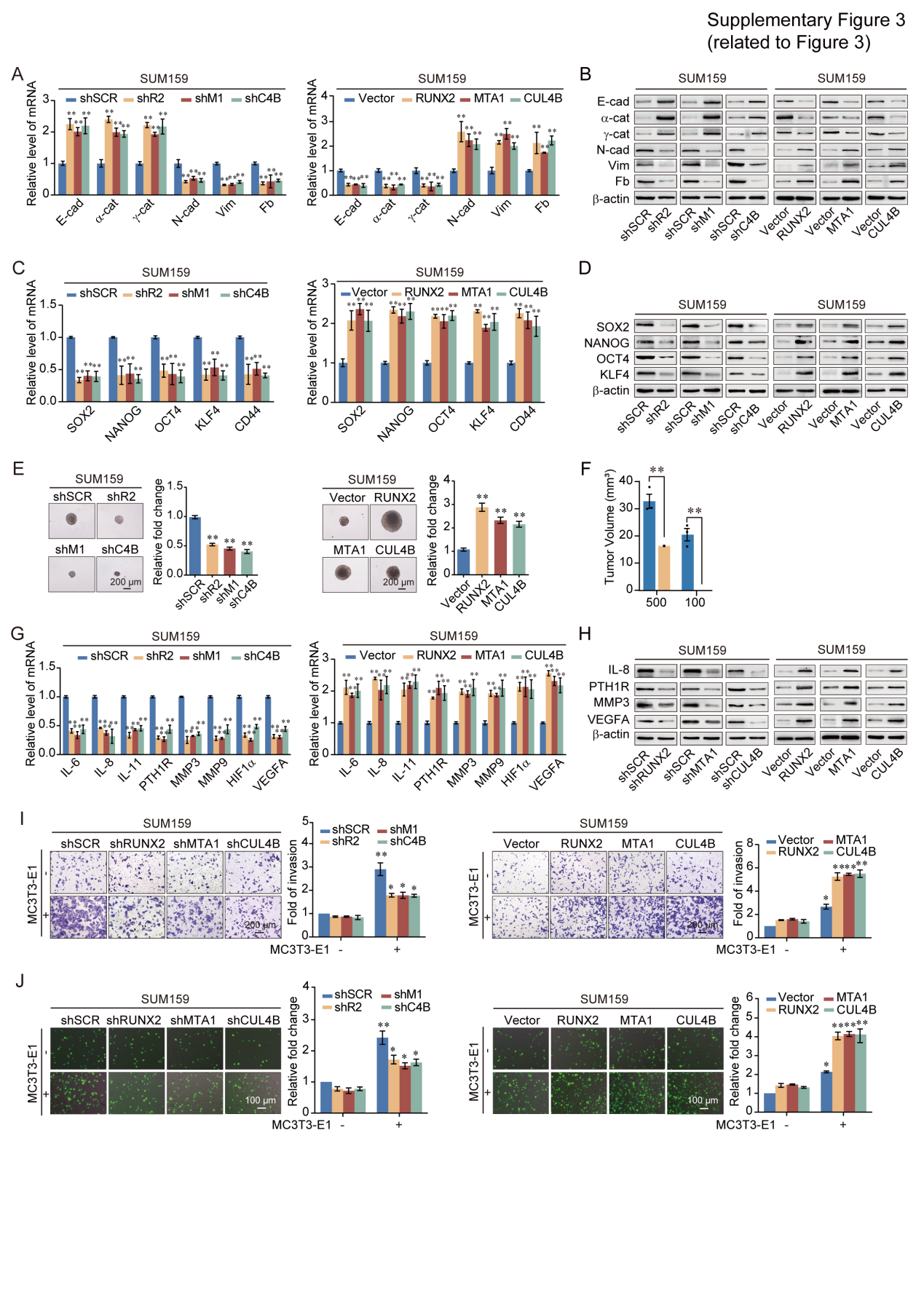


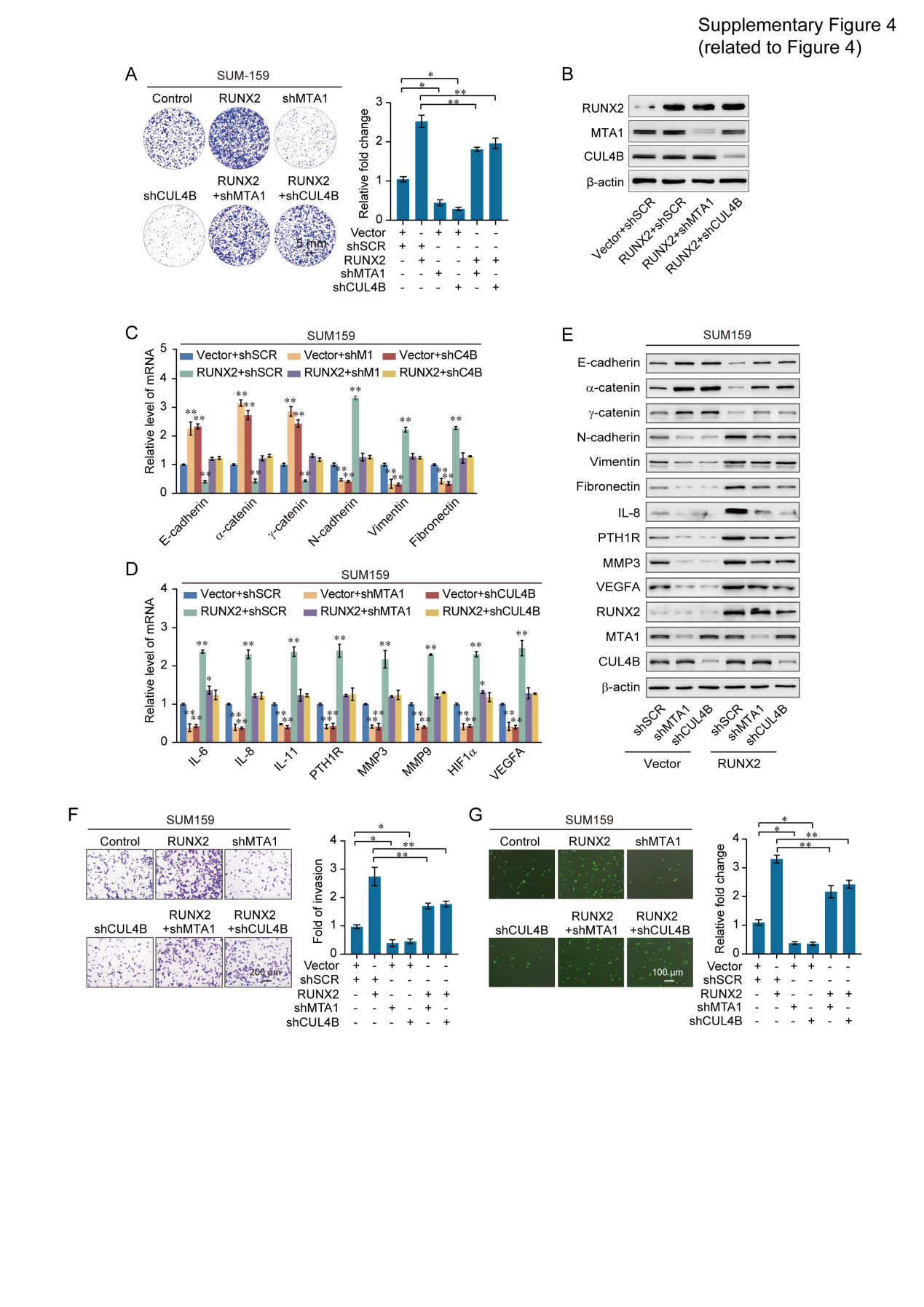


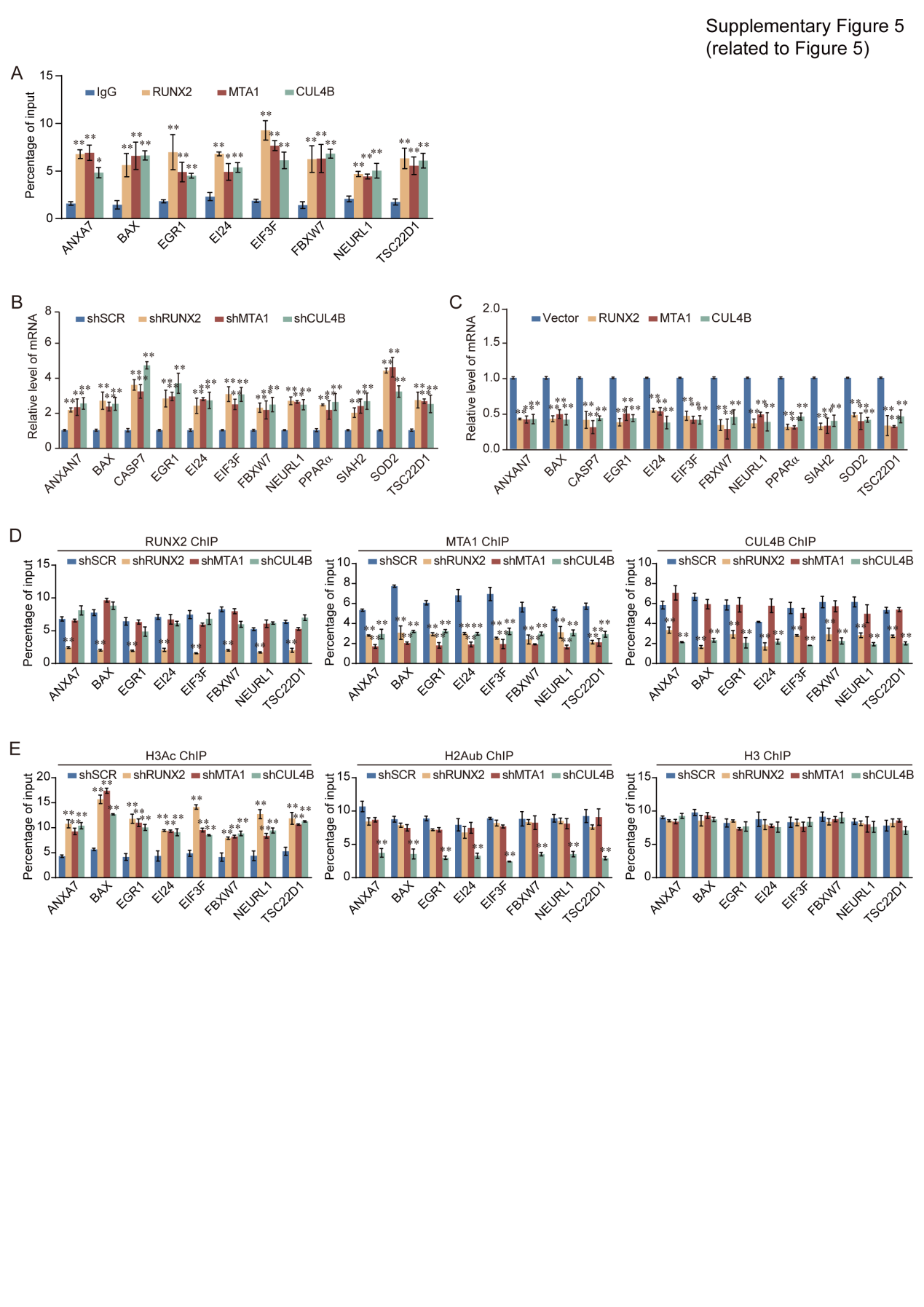


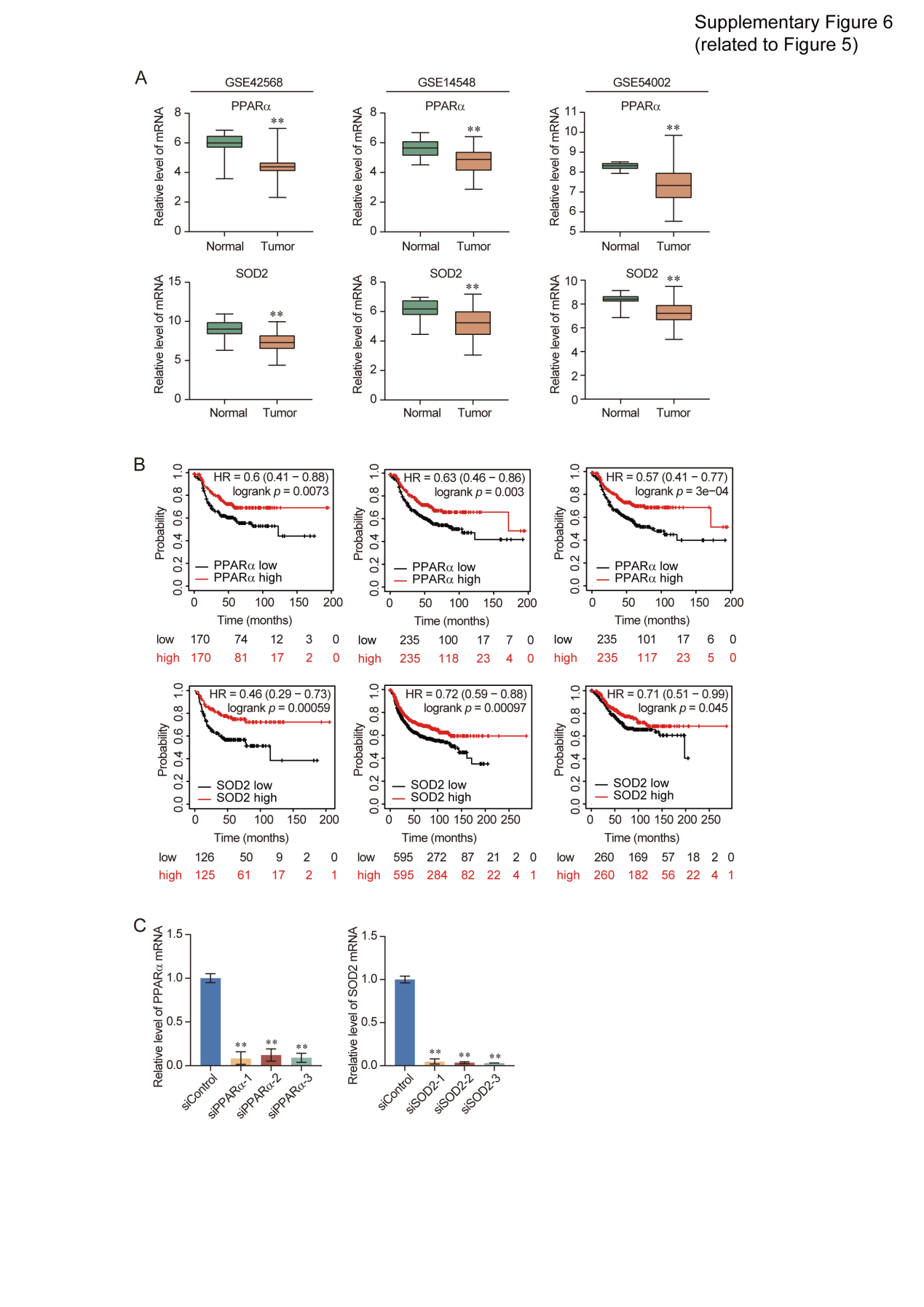


**Supplementary Figure Legends**

**Supplementary Figure 1. RUNX2 regulates tumor suppressor genes expression and participates in bone metastasis related signaling pathways.** (A) Visualized peaks at RUNX2 loci using integrative genomics viewer (IGV). (B) Volcano plot of RNA-seq results comparing siRUNX2 versus control-treated. (C) Heatmap of differential genes expression. (D) KEGG pathways analysis of downregulated and upregulated genes with depletion of RUNX2. (E) GSEA analysis of RNA-seq results. (F) Heatmap of several oncogenes and tumor suppressor genes. (G) RT-qPCR analysis for the relative mRNA expression of indicated genes in MDA-MB-231 with siRUNX2 versus control-treated. All results were presented as mean ± SD. Two-tailed unpaired *t*-test, **p* <0.05, ***p* <0.01.

**Supplementary Figure 2. The RUNX2/NuRD(MTA1)/CRL4B complex regulates the proliferation of breast cancer cells. (**A–B) Knockdown and overexpression efficiencies of RUNX2, MTA1, and CUL4B were verified by western blotting in MDA-MB-231 and SUM159 cells. (C–D) Growth curve assays were performed in MDA-MB-231 and SUM159 cells with depletion or overexpression of RUNX2, MTA1, or CUL4B. (E–F) Colony formation assays were performed in MDA-MB-231 and SUM159 cells with depletion or overexpression of RUNX2, MTA1, or CUL4B. (G–H). EdU assays were performed in MDA-MB-231 and SUM159 cells with depletion or overexpression of RUNX2, MTA1, or CUL4B. shR2, shRUNX2; shM1, shMTA1; shC4B, shCUL4B. Representative images of each group are shown. All results are presented as mean ± SD. Two-tailed unpaired *t*-test, **p* <0.05, ***p* <0.01.

**Supplementary Figure 3. RUNX2 recruits the NuRD(MTA1)/CRL4B complex to promote the proliferation, epithelial-to-mesenchymal transition, stemness, and bone metastasis of breast cancer cells.** (A) RT-qPCR results for the relative mRNA expression of epithelial and mesenchymal markers in SUM159 cells with depletion or overexpression of RUNX2, MTA1, or CUL4B. (B) Western blotting analysis of epithelial and mesenchymal markers in SUM159 cells with depletion or overexpression of RUNX2, MTA1, or CUL4B. (C) RT-qPCR results for the relative mRNA expression of stem cell markers in SUM159 cells with depletion or overexpression of RUNX2, MTA1, or CUL4B. (D) Western blotting analysis of stem cell markers in SUM159 cells with depletion or overexpression of RUNX2, MTA1, or CUL4B. (E) SUM159 cells stably knocked down or overexpressed RUNX2, MTA1, or CUL4B. Representative images of spheres were grown in suspension culture for 15 days. Cells were placed in an ultra-low attachment six-well plate (5 000/well). (F) The tumor volume of xenograft tumors. Results were presented as mean ± SEM. (G) RT-qPCR results for the relative mRNA expression of bone metastasis-related markers in SUM159 cells with depletion or overexpression of RUNX2, MTA1, or CUL4B. (H) Western blotting analysis of bone metastasis-related markers in SUM159 cells with depletion or overexpression of RUNX2, MTA1, or CUL4B. (I–J) Chemotactic migration assays (I) and cancer cell–bone matrix adhesion assays (J) of SUM159 cells with depletion or overexpression of RUNX2, MTA1, or CUL4B co-cultured with MC3T3-E1 cells. Representative images in each group were shown. shR2, shRUNX2; shM1, shMTA1; shC4B, shCUL4B; E-cad, E-cadherin; 𝛼-cat, 𝛼-catenin; 𝛾-cat, 𝛾-catenin; N-cad, N-cadherin; Vim, Vimentin; Fb, Fibronectin. All results were presented as mean ± SD. Two-tailed unpaired *t*-test, **p* <0.05, ***p* <0.01.

**Supplementary Figure 4. The RUNX2/NuRD(MTA1)/CRL4B complex promotes the proliferation and drives attraction and adhesion of breast cancer cells to bone.** (A) Colony formation assays were performed in SUM159 cells with overexpression of RUNX2 and co-transfected with shMTA1 or shCUL4B. (B) Overexpression of RUNX2 and knockdown of MTA1 and CUL4B efficiencies were verified by western blotting in MDA-MB-231 cells. (C) RT-qPCR results for the relative mRNA expression of epithelial and mesenchymal markers in SUM159 cells with overexpression of RUNX2 and co-transfected with shMTA1 or shCUL4B. (D) RT-qPCR results for the relative mRNA expression of bone metastasis-related markers in SUM159 cells with overexpression of RUNX2 and co-transfected with shMTA1 or shCUL4B. (E) Western blotting analysis of epithelial and mesenchymal and bone metastasis-related markers in SUM159 cells with overexpression of RUNX2 and co-transfected with shMTA1 or shCUL4B. (F–G) Chemotactic migration assays (F) and cancer cell–bone matrix adhesion assays (G) of SUM159 cells. These cells were co-cultured with MC3T3-E1 cells after knockdown of MTA1 or CUL4B, and overexpression of RUNX2 and co-transfected with shMTA1 or shCUL4B. shM1, shMTA1; shC4B, shCUL4B. Representative images in each group were shown. Results were presented as mean ± SD. Two-tailed unpaired *t*-test, **p* <0.05, ***p* <0.01.

**Supplementary Figure 5. RUNX2 recruits the NuRD(MTA1)/CRL4B complex for transcriptional repression in breast cancer cells.** (A) Verification of ChIP-seq results using qChIP analysis of indicated genes. (B–C) RT-qPCR results for the relative mRNA expression of ChIP-seq indicated genes in MDA-MB-231 cells with depletion (B) or overexpression (C) of RUNX2, MTA1, or CUL4B. (D–E) MDA-MB-231 cells were infected with lentiviruses carrying the indicated shRNAs. qChIP analysis of the target gene promoters was performed using antibodies against RUNX2, MTA1, or CUL4B (D) or against histone H3Ac, H2AK119ub1 (E); Histone H3 was detected as an internal control. Results were represented as fold change over control with β-actin as a negative control. H3Ac, pan-H3 acetylation; H2Aub, H2AK119 monoubiquitination. All results were presented as mean ± SD. Two-tailed unpaired *t*-test, **p* <0.05, ***p* <0.01.

**Supplementary Figure 6.** **The expression of PPARα/SOD2 is decreased in breast cancer and positively with a better overall survival.** (A) PPARα and SOD2 expression in breast cancer microarray datasets were obtained from GEO datasets (GSE42568, GSE14548, and GSE54002). Results were presented as mean ± SEM. (B) Kaplan–Meier survival analysis of the relationship between survival time and PPARα or SOD2 signatures in breast cancer using the online tool (http://kmplot.com/analysis/). (C) The knockdown efficiencies of PPARα and SOD2 were verified by RT-qPCR in MDA-MB-231 cells. Results were presented as mean ± SD. Two-tailed unpaired *t*-test, **p* <0.05, ***p* <0.01.
