# Supplementary material for: RUNX2 recruits the NuRD(MTA1)/CRL4B complex to promote breast cancer progression and bone metastasis
Source: Cell Death Differ. 2022 May 9;29(11):2203–17. doi: 10.1038/s41418-022-01010-2 (PMC9613664; doi:10.1038/s41418-022-01010-2)
Supplement: Supplementary file 2 — Supplementary material file Tables [file 41418_2022_1010_MOESM2_ESM.docx]

**Supplementary Table S1.** siRNA sequences

| **siRNA sequences** | | |
| --- | --- | --- |
| **Genes** | **Sense (5'-3')** | **Antisense (5'-3')** |
| siControl | UUCUCCGAACGUGUCACGUTT | ACGUGACACGUUCGGAGAATT |
| siRUNX1-1 | CCAGGUUGCAAGAUUUAAUTT | AUUAAAUCUUGCAACCUGGTT |
| siRUNX1-2 | GGCAGAAACUAGAUGAUCATT | UGAUCAUCUAGUUUCUGCCTT |
| siRUNX1-3 | GGAUCCAUUGCCUCUCCUUTT | AAGGAGAGGCAAUGGAUCCTT |
| siRUNX2-1 | GGUCCUAUGACCAGUCUUATT | UAAGACUGGUCAUAGGACCTT |
| siRUNX2-2 | CCAGCCACCUUUACUUACATT | UGUAAGUAAAGGUGGCUGGTT |
| siRUNX2-3 | CACGCUAUUAAAUCCAAAUTT | AUUUGGAUUUAAUAGCGUGTT |
| siRUNX3-1 | UGACGAGAACUACUCCGCUTT | AGCGGAGUAGUUCUCGUCATT |
| siRUNX3-2 | CCCUGACCAUCACUGUGUUTT | AACACAGUGAUGGUCAGGGTT |
| siRUNX3-3 | CCUCGGAACUGAACCCAUUTT | AAUGGGUUCAGUUCCGAGGTT |
| siPPARα-1 | GGAGCAUUGAACAUCGAAUTT | AUUCGAUGUUCAAUGCUCCTT |
| siPPARα-2 | GCAGGAGGGUAUUGUACAUTT | AUGUACAAUACCCUCCUGCTT |
| siPPARα-3 | GGGUUUAUAACUCGUGAAUTT | AUUCACGAGUUAUAAACCCTT |
| siSOD2-1 | GGGUUGGCUUGGUUUCAAUTT | AUUGAAACCAAGCCAACCCTT |
| siSOD2-2 | GGAGAAUGUAACUGAAAGATT | UCUUUCAGUUACAUUCUCCTT |
| siSOD2-3 | GGUGGUCAUAUCAAUCAUATT | UAUGAUUGAUAUGACCACCTT |

**Supplementary Table S2.** shRNA sequences

| **shRNA sequences** | |
| --- | --- |
| shSCR | TTCTCCGAACGTGTCACGT |
| shCULB | GGATTCATTGGATAGCGTTCT |
| shMTA1 | GGCTAACTTATTCCGAGAATG |
| shRUNX2 | AAGGTTCAACGATCTGATTTG |

**Supplementary Table S3.** Primers used for RT-qPCR

| **The Primers used in quantitative real-time PCR (qPCR)** | | |
| --- | --- | --- |
| **Genes** | **Sense (5'-3')** | **Antisense (5'-3')** |
| RUNX1 | TGAGCTGAGAAATGCTACCGC | ACTTCGACCGACAAACCTGAG |
| RUNX2 | TGGTTACTGTCATGGCGGGTA | TCTCAGATCGTTGAACCTTGCTA |
| RUNX3 | AGCACCACAAGCCACTTCAG | GGGAAGGAGCGGTCAAACTG |
| IL-6 | CCTTCGGTCCAGTTGCCTTCT | CAGTGCCTCTTTGCTGCTTT |
| IL-8 | CTTGGCAGCCTTCCTGATTT | ACAACCCTCTGCACCCAGTT |
| IL-11 | ACATGAACTGTGTTTGCCGC | ATCTGGCTTTGGAAGGACGG |
| PTH1R | AGTGCGAAAAACGGCTCAAG | GATGCCTTATCTTTCCTGGGC |
| MMP3 | CTGGACTCCGACACTCTGGA | CAGGAAAGGTTCTGAAGTGACC |
| MMP9 | CAGTCCACCCTTGTGCTCTTCC | CTGCCACCCGAGTGTAACCAT |
| HIF1α | GAACGTCGAAAAGAAAAGTCTCG | CCTTATCAAGATGCGAACTCACA |
| VEGFA | AGCCTTGCCTTGCTGCTCTA | CACCAGGGTCTCGATTGGAT |
| E-cadherin | CATTTCTTGGTCTACGCCTG | GAGAGGAGTTGGGAAATGTG |
| α-catennin | AGCTGAAAGTTGTGGAAGAT | CCAACATCTTTCAATTCCTGTTG |
| γ-catennin | GGACAAGAACCCAGACTACC | GTGGCATCCATGTCATCTCC |
| Fibronectin | CCATCCATTGATTTAACCAACTT | TACCAGGCAGGAGATTTGTTAA |
| N-cadherin | CACTGCTCAGGACCCAGAT | TAAGCCGAGTGATGGTCC |
| Vimentin | ATTGAGATTGCCACCTACAG | ATCCAGATTAGTTTCCCTCAG |
| KLF4 | CCCACATGAAGCGACTTCCC | CAGGTCCAGGAGATCGTTGAA |
| OCT4 | ATCACCCTGGGATATACACAG | CTGCTTTGCATATCTCCTGA |
| CD44 | CTGCCGCTTTGCAGGTGTA | CATTGTGGGCAAGGTGCTATT |
| SOX2 | GCCGAGTGGAAACTTTTGTCG | GGCAGCGTGTACTTATCCTTCT |
| NANOG | CCCCAGCCTTTACTCTTCCTA | CCAGGTTGAATTGTTCCAGGTC |
| FADD | GCTGGCTCGTCAGCTCAAA | ACTGTTGCGTTCTCCTTCTCT |
| HSP90B1 | GCTGACGATGAAGTTGATGTGG | CATCCGTCCTTGATCCTTCTCTA |
| TNFAIP3 | TCCTCAGGCTTTGTATTTGAGC | TGTGTATCGGTGCATGGTTTTA |
| CDK1 | AAACTACAGGTCAAGTGGTAGCC | TCCTGCATAAGCACATCCTGA |
| CHEK1 | ATATGAAGCGTGCCGTAGACT | TGCCTATGTCTGGCTCTATTCTG |
| ENO2 | TCATGGTGAGTCATCGCTCAGGAG | ATGTCCGGCAAAGCGAGCTTCATC |
| GAM1 | GGAAACGTGTACTGATTGCAGCCC | TTCCATGGCTTTGCGCACCGTCT |
| CCND1 | TGAGGGACGCTTTGTCTGTC | CTTCTGCTGGAAACATGCCG |
| CDC23 | CATGGCTGCAATAGCAAGAAAG | CGCCTCATTTTTCACTTGTCCT |
| GAPDH | GTCAACGGATTTGGTCGTAT | GAACATGTAAACCATGTAGTTGA |
| LDHA | ATGGCAACTCTAAAGGATCA | GCAACTTGCAGTTCGGGC |
| STMN1 | TCAGCCCTCGGTCAAAAGAAT | TTCTCGTGCTCTCGTTTCTCA |
| SOD2 | TGGACAAACCTCAGCCCTAAC | AGCCTTGGACACCAACAGATG |
| CAPS7 | AGTGACAGGTATGGGCGTTC | CGGCATTTGTATGGTCCTCTT |
| PPARα | TTCGCAATCCATCGGCGAG | CCACAGGATAAGTCACCGAGG |
| BAX | CCCGAGAGGTCTTTTTCCGAG | CCAGCCCATGATGGTTCTGAT |
| SIAH2 | CGCCAGAAGTTGAGCTGCT | TGGTGGCATACTTACAGGGAA |
| ANXA7 | AGGCCAAGGATTTGGAGTCC | TGACTAGGGTAAGTAGGTTGTCC |
| FBXW7 | CGACGCCGAATTACATCTGTC | CGTTGAAACTGGGGTTCTATCA |
| EIF3F | ACGGGCCATGACATCACAG | AAGTGCTGACGTAGGCTTTGA |
| EI24 | TGCCAGAGGAATCAAAGACTCC | TCTCTTGCTTCCGCTCTATACT |
| TSC22D1 | AGCGTCAGGTCCCGTTTTC | CTGGTGCATTGTGTTGGGT |
| EGR1 | GGTCAGTGGCCTAGTGAGC | GTGCCGCTGAGTAAATGGGA |
| NEURL1 | TCGGCTGTTATGCTGTTCTTC | GAGTGAGTTCTGCGGGATGG |
| Actin | CATGTACGTTGCTATCCAGGC | CCTTATCAAGATGCGAACTCACA |
| Mouse cathepsinK | AAGTGGTTCAGAAGATGACGGGAC | TCTTCAGAGTCAATGCCTCCGTTC |
| Mouse NFATc1 | TGGAGAAGCAGAGCACAGAC | GCGGAAAGGTGGTATCTCAA |
| Mouse C-fos | CAAGCGGAGACAGATCAACTTG | TTTCCTTCTCTTTCAGCAGATTGG |
| Mouse TRAP | GCTGGAAACCATGATCACCT | GAGTTGCCACACAGCATCAC |
| Mouse Actin | GTACGCCAACACAGTGCT | CGTCATACTCCTGCTTGCTG |

**Supplementary Table S4.** Primers used for qChIP

| **The Primers used in ChIP Assays** | | |
| --- | --- | --- |
| **Genes** | **Sense (5'-3')** | **Antisense (5'-3')** |
| ANXA7 | TATTGATCTGAGCCCTCGTAA | CCTCTGAAATACCGTCCTCC |
| BAX | TTAGTCATCTATAACGTCCTGC | CATCTCCCGATAAGTGCC |
| CAPS7 | TCCGAGTTGATGTGAAGGC | CCAAGTGACCCAAAGAGC |
| EGR1 | GAGGGAAGAAGGCGGAGGGA | TTGAAGGGTCTGGAACGGCAC |
| EI24 | AGCGTTGTGGAGTCAATGGG | CGACGGAGACTGGGAAATGTAG |
| EIF3F | TTACCAACGGCGGGACTA | GAAGCGGAAATGACAGCA |
| FBXW7 | CGGTAGGATGACAGTGAA | CAGGGAACCCGTAAGAAC |
| NEURL1 | GGGGAACCTTGGAACATC | GCAGCCTATGGCAGTCAA |
| PPARA | ACCTCCCGCCACCTGTTT | AGCCCGGCTCTGCTACTCT |
| SIAH2 | GTTGGCTCAAGCAAAGTC | CGTAGGTTGGGTCGT |
| SOD2 | AAGCCCAGCCCTTCCTGTT | GGTGCTGAACCGTTTCCGT |
| TSC22D1 | TAAGGTTTGTGGCTCTACGG | AGTCCCACCACTGTAA |
| Actin | CCAGCACCCCAAGGCG | GGCTTCGCCGCACAGT |

**Supplementary Table S5**. Clinical traits of patients related to Figure 1E

| **Clinical Characteristics** | Age | TNM | ER Status | PR Status | HER2 status | Ki67 | Grade |
| --- | --- | --- | --- | --- | --- | --- | --- |
| Patient 1 | 67 | T2N1aM0 | - | - | + | 50% | G3 |
| Patient 2 | 63 | T2N0M0 | + | + | - | 10% | Not applicable |
| Patient 3 | 56 | T1cN2aM0 | + | + | - | 10% | G2 |
| Patient 4 | 75 | T1miN0M0 | + | + | - | 15% | G1 |
| Patient 5 | 57 | T1miN0M0 | - | - | + | 15% | Not applicable |
| Patient 6 | 63 | T3N3M0 | + | + | - | 25% | G2 |

**Supplementary Table S6.** Mass spectrometry results of RUNX2-containing protein complex in MDA-MB-231 cells

| **Gene Symbol** | **Gene ID** | **Score** | **Peptides** | **Coverage (%)** |
| --- | --- | --- | --- | --- |
| RUNX2 | 860 | 35.68 | 56 | 27.86 |
| CCT3 | 7203 | 20.32 | 10 | 18.90 |
| PRDX1 | 5052 | 15.45 | 17 | 60.82 |
| PARP1 | 142 | 32.21 | 12 | 17.26 |
| MTA1 | 9112 | 11.71 | 20 | 11.84 |
| EEF2 | 1938 | 15.80 | 10 | 19.00 |
| STUB1 | 10273 | 9.51 | 7 | 27.06 |
| USP7 | 7874 | 14.29 | 9 | 11.16 |
| MTHFD1 | 4522 | 13.29 | 6 | 16.58 |
| MCM3 | 4172 | 10.80 | 6 | 16.34 |
| RBM10 | 8241 | 10.20 | 6 | 8.54 |
| DDB1 | 1642 | 10.19 | 5 | 8.86 |
| EIF4B | 1975 | 8.81 | 5 | 16.88 |
| PRMT5 | 10419 | 8.72 | 5 | 11.64 |
| PPM1B | 5495 | 6.90 | 4 | 15.08 |
| BAG3 | 9531 | 6.14 | 3 | 14.09 |
| CBFB | 865 | 2.06 | 9 | 18.72 |
| CSDE1 | 7812 | 4.79 | 2 | 9.88 |
| Cyclin B1 | 891 | 2.79 | 3 | 9.52 |
| H3F3B | 3021 | 10.22 | 3 | 26.52 |
| HSPA9 | 3313 | 3.27 | 2 | 6.627 |
| EFTUD2 | 9343 | 4.95 | 3 | 3.909 |
| RbAp46 | 5931 | 2.04 | 2 | 6.353 |
| HDAC2 | 3066 | 1.55 | 1 | 20.00 |
| H3F3B | 3021 | 5.05 | 3 | 26.52 |
