# Supplementary material for: RUNX2 recruits the NuRD(MTA1)/CRL4B complex to promote breast cancer progression and bone metastasis
Source: Cell Death Differ. 2022 May 9;29(11):2203–17. doi: 10.1038/s41418-022-01010-2 (PMC9613664; doi:10.1038/s41418-022-01010-2)
Supplement: Supplementary file 3 — Supplementary material file Legends [file 41418_2022_1010_MOESM3_ESM.docx]

**Figure legends**

**Figure 1. Upregulation of RUNX2 is correlated with breast cancer progression.** (A) Analysis of RUNX family expression in normal and breast cancer by using GEO and TCGA database. (B) Analysis of RUNX family expression in breast cancer by using GEO database. (C) Kaplan–Meier survival analysis of the relationship between survival time and expression of RUNX family using an online tool (http://kmplot.com/analysis/). (D) Expression of RUNX family in normal and breast cancer cell lines. (E) Expression of RUNX family in breast cancer and adjacent tissues (*n* = 6). Results were presented as mean ± SEM. (F) Knockdown efficiencies of RUNX1, RUNX2, and RUNX3 were verified by RT-qPCR. (G) Growth curve assays were performed in MDA-MB-231 and SUM159 cells transfected with siRNA against RUNX1, RUNX2, and RUNX3. (H) EdU assays were performed in RUNX family-deficient MDA-MB-231 and SUM159 cells and corresponding statistical analysis. Representative images in each group were shown. Results were presented as mean ± SD. Two-tailed unpaired *t*-test, **p* <0.05, ***p* <0.01.

**Figure 2. RUNX2 is physically associated with the NuRD(MTA1) complex and the CRL4B complex.** (A) Immunoaffinity purification and mass spectrometry analysis of RUNX2-containing protein complexes. Whole-cell extracts from MDA-MB-231 cells stably expressing FLAG-Vector or FLAG-RUNX2 were immunopurified using anti-FLAG affinity columns and eluents with FLAG peptide. Elutes were resolved using SDS-PAGE and silver-stained. Protein bands were retrieved and analyzed using mass spectrometry. (B) Western blotting analysis of the purified fractions using antibodies against FLAG in MDA-MB-231 cells. (C) Immunoprecipitation with antibodies against RUNX2 followed by immunoblotting with antibodies against MTA1, MTA2, MTA3, HDAC1, HDAC2, RbAp46/48, MBD3, CUL4B, DDB1, and ROC1. (D) Immunoprecipitation with antibodies against MTA1, MTA2, MTA3, HDAC1, HDAC2, RbAp46/48, MBD3, CUL4B, DDB1, and ROC1 followed by immunoblotting with antibodies against RUNX2. (E–F) GST pull-down assays with GST-fused proteins expressed in bacteria and *in vitro* translated proteins as indicated. (G–I) Identification of the essential domains of RUNX2, MTA1, or CUL4B required for interaction. (J) Schematic diagram depicting molecular interactions among RUNX2/NuRD(MTA1)/CRL4B complex. IB, immunoblotting; aa, amino acid.

**Figure 3. RUNX2 recruits the NuRD(MTA1)/CRL4B complex to promote the epithelial-to-mesenchymal transition, stemness, and bone metastasis of breast cancer cells.** (A) RT-qPCR results for the relative mRNA expression of epithelial and mesenchymal markers in MDA-MB-231 cells with depletion or overexpression of RUNX2, MTA1, or CUL4B. (B) Western blotting analysis of epithelial and mesenchymal markers in MDA-MB-231 cells with depletion or overexpression of RUNX2, MTA1, or CUL4B. (C) RT-qPCR results for the relative mRNA expression of stem cell markers in MDA-MB-231 cells with depletion or overexpression of RUNX2, MTA1, or CUL4B. (D) Western blotting analysis of stem cell markers in MDA-MB-231 cells with depletion or overexpression of RUNX2, MTA1, or CUL4B. (E) MDA-MB-231 cells stably knocked down or overexpressed RUNX2, MTA1, or CUL4B. Representative images of spheres were grown in suspension culture for 15 days. Cells were placed in an ultra-low attachment six-well plate (5 000/well). (F) Tumorigenicity was tested by injecting MDA-MB-231-shSCR or MDA-MB-231-shRUNX2 cells into the mammary gland fat pads of NOD/SCID mice at various dilutions (*n* = 5 in each group). The stem cell frequency in xenograft tumors was calculated using the Extreme Limiting Dilution Analysis (ELDA) software (http://bioinf.wehi.edu.au/software/elda/index.html), results were presented as mean ± SEM. (G) RT-qPCR results for the relative mRNA expression of bone metastasis-related markers in MDA-MB-231 cells with depletion or overexpression of RUNX2, MTA1, or CUL4B. (H) Western blotting analysis of bone metastasis-related markers in MDA-MB-231 cells with depletion or overexpression of RUNX2, MTA1, or CUL4B. (I-J) RT-qPCR results for the relative mRNA expression of osteoclastogenesis markers in RAW264.7 cells co-cultured with the conditional medium of MDA-MB-231 and SUM159 cells with depletion or overexpression of RUNX2, MTA1, or CUL4B. (K–L) Chemotactic migration assays (K) and cancer cell–bone matrix adhesion assays (L) of MDA-MB-231 cells with depletion or overexpression of RUNX2, MTA1, or CUL4B co-cultured with MC3T3-E1 cells. Representative images in each group were shown. shR2, shRUNX2; shM1, shMTA1; shC4B, shCUL4B; E-cad, E-cadherin; 𝛼-cat, 𝛼-catenin; 𝛾-cat, 𝛾-catenin; N-cad, N-cadherin; Vim, Vimentin; Fb, Fibronectin. All results were presented as mean ± SD. Two-tailed unpaired *t*-test, **p* <0.05, ***p* <0.01.

**Figure 4. The RUNX2/NuRD(MTA1)/CRL4B complex promotes proliferation and drives the attraction and adhesion of breast cancer cells to bone.** (A) Colony formation assays were performed in MDA-MB-231 cells with overexpression of RUNX2 and co-transfected with shMTA1 or shCUL4B. (B) MDA-MB-231 cells infected with lentiviruses carrying the indicated expression constructs and/or shRNAs were inoculated orthotopically into the abdominal mammary fat pad of 6-week-old female NOD/SCID mice (*n* = 3); the tumor volume: length × width²; Results were presented as mean ± SEM. (C) RT-qPCR results for the relative mRNA expression of epithelial and mesenchymal markers in MDA-MB-231 cells with overexpression of RUNX2 and co-transfected with shMTA1 or shCUL4B. (D) RT-qPCR results for the relative mRNA expression of bone metastasis-related markers in MDA-MB-231 cells with overexpression of RUNX2 and co-transfected with shMTA1 or shCUL4B. (E) Western blotting analysis of epithelial and mesenchymal and bone metastasis-related markers in MDA-MB-231 cells with overexpression of RUNX2 and co-transfected with shMTA1 or shCUL4B. (F) RT-qPCR results for the relative mRNA expression of osteoclastogenesis markers in RAW264.7 cells co-cultured with the conditional medium of MDA-MB-231 and SUM159 cells with overexpression of RUNX2 and depletion of MTA1 or CUL4B. (G–H) Chemotactic migration assays (G) and cancer cell–bone matrix adhesion assays (H) of MDA-MB-231 cells. These cells were co-cultured with MC3T3-E1 cells after knockdown of shMTA1 or CUL4B, and overexpression of RUNX2 and co-transfected with MTA1 or shCUL4B. shM1, shMTA1; shC4B, shCUL4B. Representative images in each group were shown. Results were presented as mean ± SD. Two-tailed unpaired *t*-test, **p* <0.05, ***p* <0.01.

**Figure 5. RUNX2 recruits the NuRD(MTA1)/CRL4B complex for transcriptional repression in breast cancer cells.** (A) Genomic distribution of RUNX2 and MTA1 determined using ChIP-seq analysis. (B) ChIP-seq density heatmaps of RUNX2 and MTA1. (C) RUNX2 and MTA1-bound motifs analyzed using the MEME suite. (D) Venn diagram of overlapping promoters bound by RUNX2/MTA1/CUL4B complex. (E) KEGG pathways analysis of 3 857 unique genes. (F) Verification of ChIP-seq results using qChIP analysis of indicated genes. (G–H) MDA-MB-231 cells were infected with lentiviruses carrying the indicated shRNAs. qChIP analysis of the target gene promoters was performed using antibodies against RUNX2, MTA1, or CUL4B (G) or against histone H3Ac, H2AK119ub1 (H); Histone H3 was detected as an internal control. Results were represented as fold change over control with β-actin as a negative control. (I) ChIP and Re-ChIP experiments in MDA-MB-231 cells with the indicated antibodies. (J) Knockdown and overexpression efficiencies of RUNX2, MTA1, or CUL4B verified by RT-qPCR. (K–L) Western blotting analysis of ChIP-seq indicated genes (PPARα and SOD2) in MDA-MB-231 cells with depletion or overexpression of RUNX2, MTA1, or CUL4B. TSS, transcriptional start site; shR2, shRUNX2; shM1, shMTA1; shC4B, shCUL4B; R2, RUNX2; M1, MTA1; C4B, CUL4B; H3Ac, pan-H3 acetylation; H2Aub, H2AK119 monoubiquitination; 1st round Abs, first round antibodies. All results were presented as mean ± SD. Two-tailed unpaired *t*-test, **p* <0.05, ***p* <0.01.

**Figure 6. RUNX2 promotes the invasion and drives attraction and adhesion of breast cancer cells to the bone by inhibiting PPARα/SOD2 expressions.** (A–C) Expression of the indicated epithelial and mesenchymal markers (A) and bone metastasis-related markers (B) were measured by RT-qPCR or western blotting (C) in MDA-MB-231 and SUM159 cells with depletion of RUNX2 and PPARα, or depletion of RUNX2 and SOD2. (D) RT-qPCR results for the relative mRNA expression of osteoclastogenesis markers in RAW264.7 cells co-cultured with the conditional medium of MDA-MB-231 and SUM159 cells with depletion and overexpression of RUNX2 and PPARα, or depletion of RUNX2 and SOD2. (E–F) Chemotactic migration assays (E) and cancer cell–bone matrix adhesion assays (F) of MDA-MB-231 and SUM159 cells. These cells were co-cultured with MC3T3-E1 cells after depletion of RUNX2 and PPARα or SOD2. siCon, siControl; shR2, shRUNX2; siP, siPPARα; siS2, siSOD2; E-cad, E-cadherin; α-cat, α-catenin; γ-cat, γ-catenin; N-cad, N-cadherin; Vim, Vimentin; Fb, Fibronectin. Representative images in each group are shown. All results are presented as mean ± SD. Two-tailed unpaired *t*-test, **p* <0.05, ***p* <0.01.

**Figure 7. The expression of RUNX2 is upregulated in multiple carcinomas and is the potential cancer biomarker.** (A) Immunohistochemical staining of RUNX2 and PPARα in breast cancer and adjacent tissues (*n* = 10). (B) Analysis of published clinical datasets (GSE48390) for the expression of RUNX2, MTA1, CUL4B, PPARα, and SOD2 by two-tailed unpaired *t*-test. (C) Kaplan–Meier survival analysis of GSE3494 for the relationship between survival time and RUNX2/PPARα, MTA1/PPARα, and CUL4B/PPARα expression signatures in breast cancer. Survival curves were calculated using Kaplan–Meier method. Log-rank tests were used for the statistical analysis. (D) Analysis of a GEO dataset (GSE14017) for the expression of RUNX2 in breast cancer with bone, brain, or lung metastasis. (E) Immunohistochemical staining of RUNX2 in paired tumor tissues of thyroid, rectum, liver, and lung versus adjacent normal tissues (*n* = 6). (F) RUNX2 expression in gastric and lung cancers microarray datasets available from GAPIA (https://gepia.cancer-pku.cn/). (G) Kaplan–Meier survival analysis of the relationship between survival time and RUNX2 signature in gastric and lung cancers using the online tool (http://kmplot.com/analysis/). (H) The proposed regulatory mechanisms of the RUNX2/MTA1/CUL4B complex in controlling invasion and bone metastasis of breast cancer. All results were presented as mean ± SEM. Two-tailed unpaired *t*-test, **p* <0.05, ***p* <0.01.
