# Supplementary material for: RUNX2 recruits the NuRD(MTA1)/CRL4B complex to promote breast cancer progression and bone metastasis
Source: Cell Death Differ. 2022 May 9;29(11):2203–17. doi: 10.1038/s41418-022-01010-2 (PMC9613664; doi:10.1038/s41418-022-01010-2)

Uncropped blots related to Figure 2A-B

Figure 2A

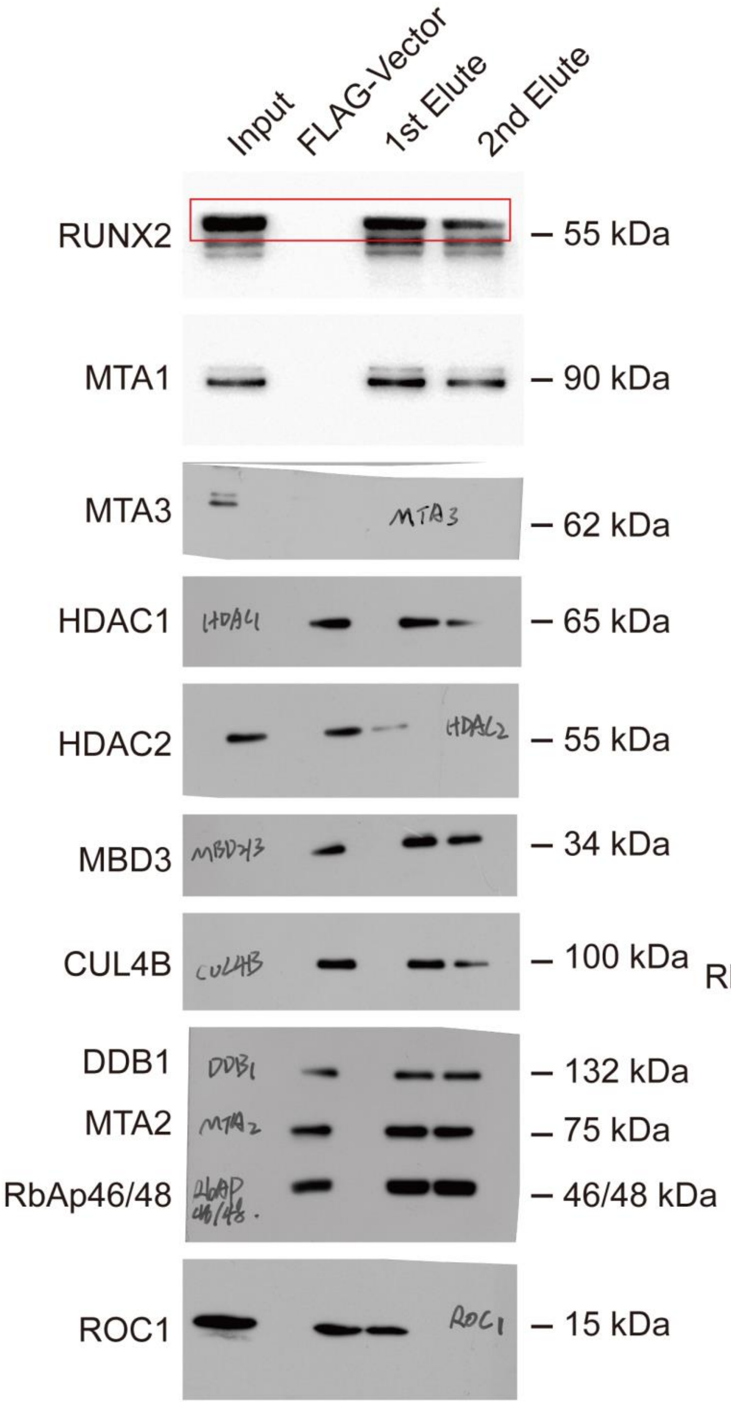

Figure 2B

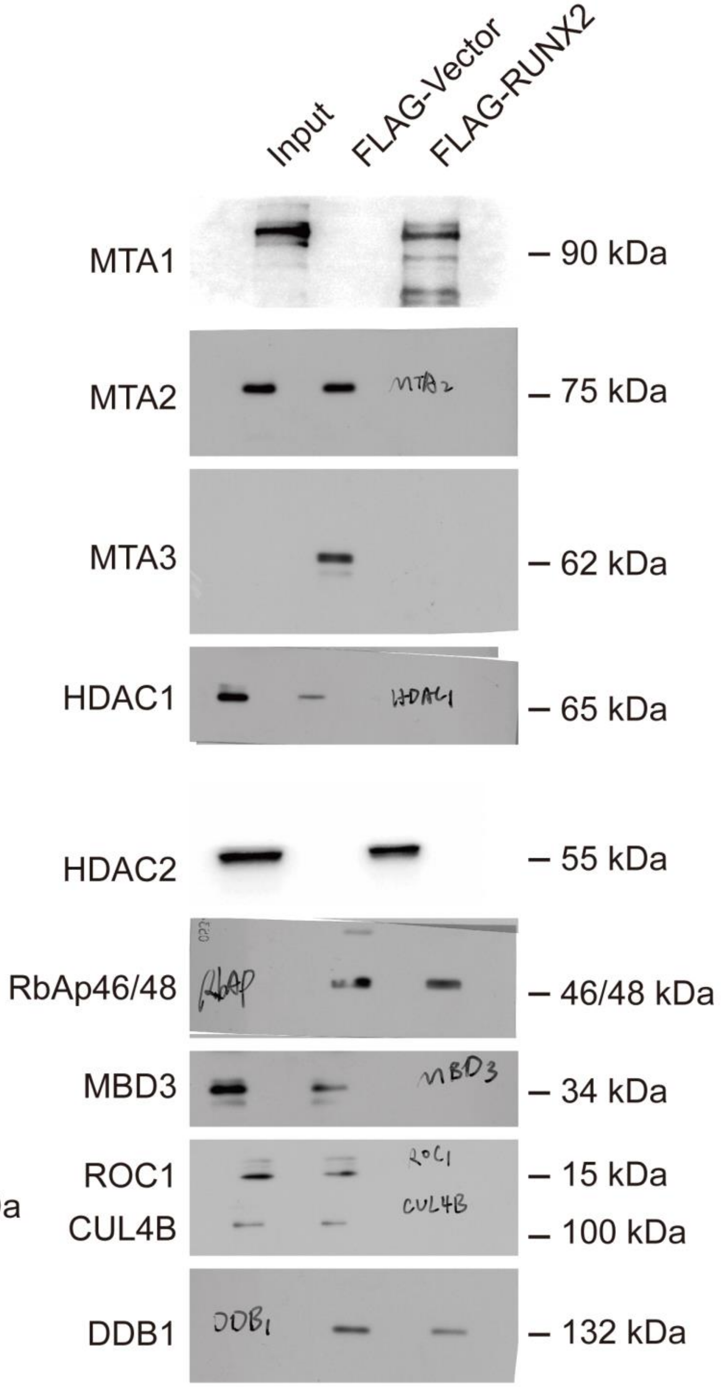

Uncropped blots related to Figure 2C

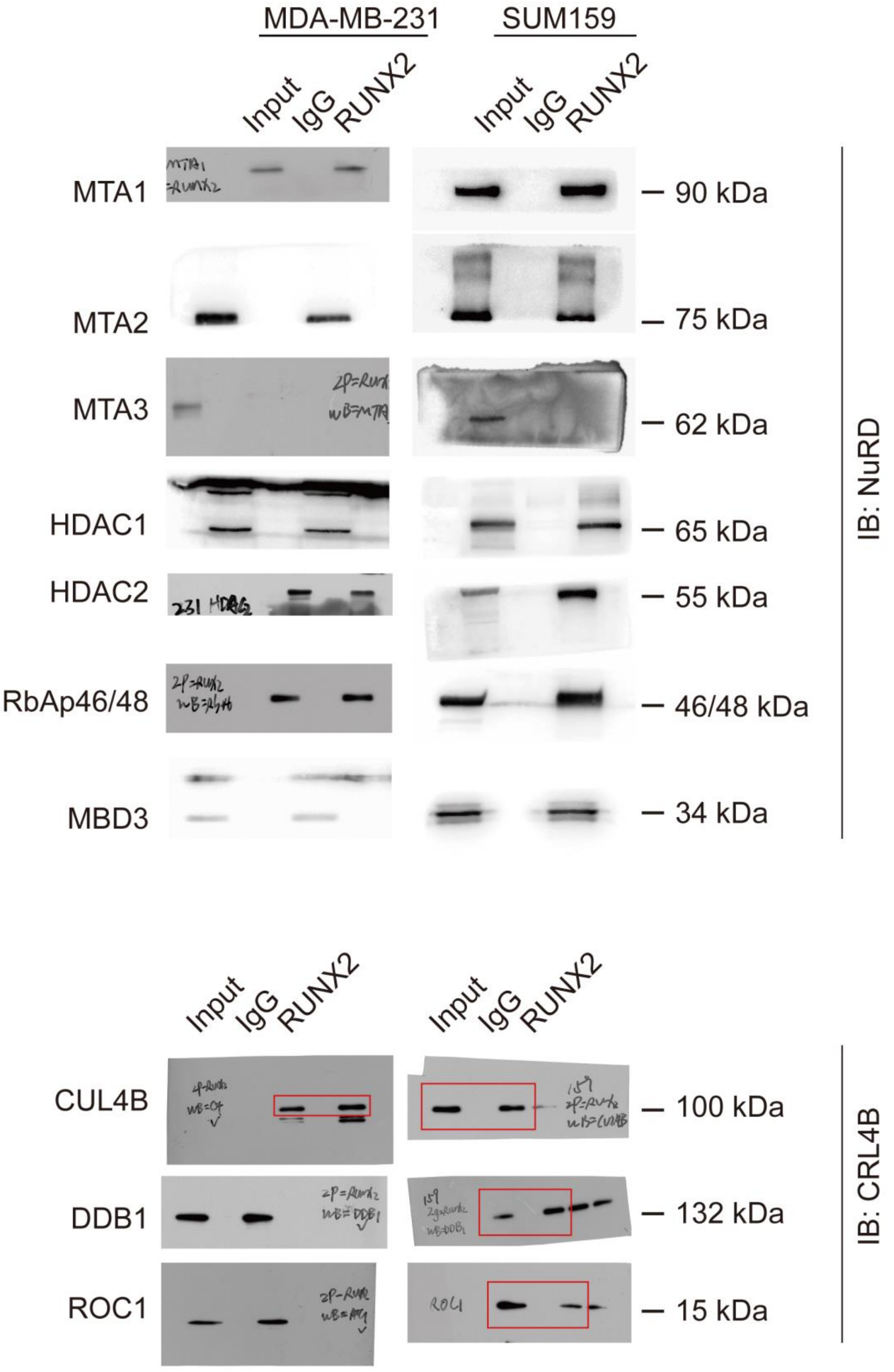

Uncropped blots related to Figure 2D

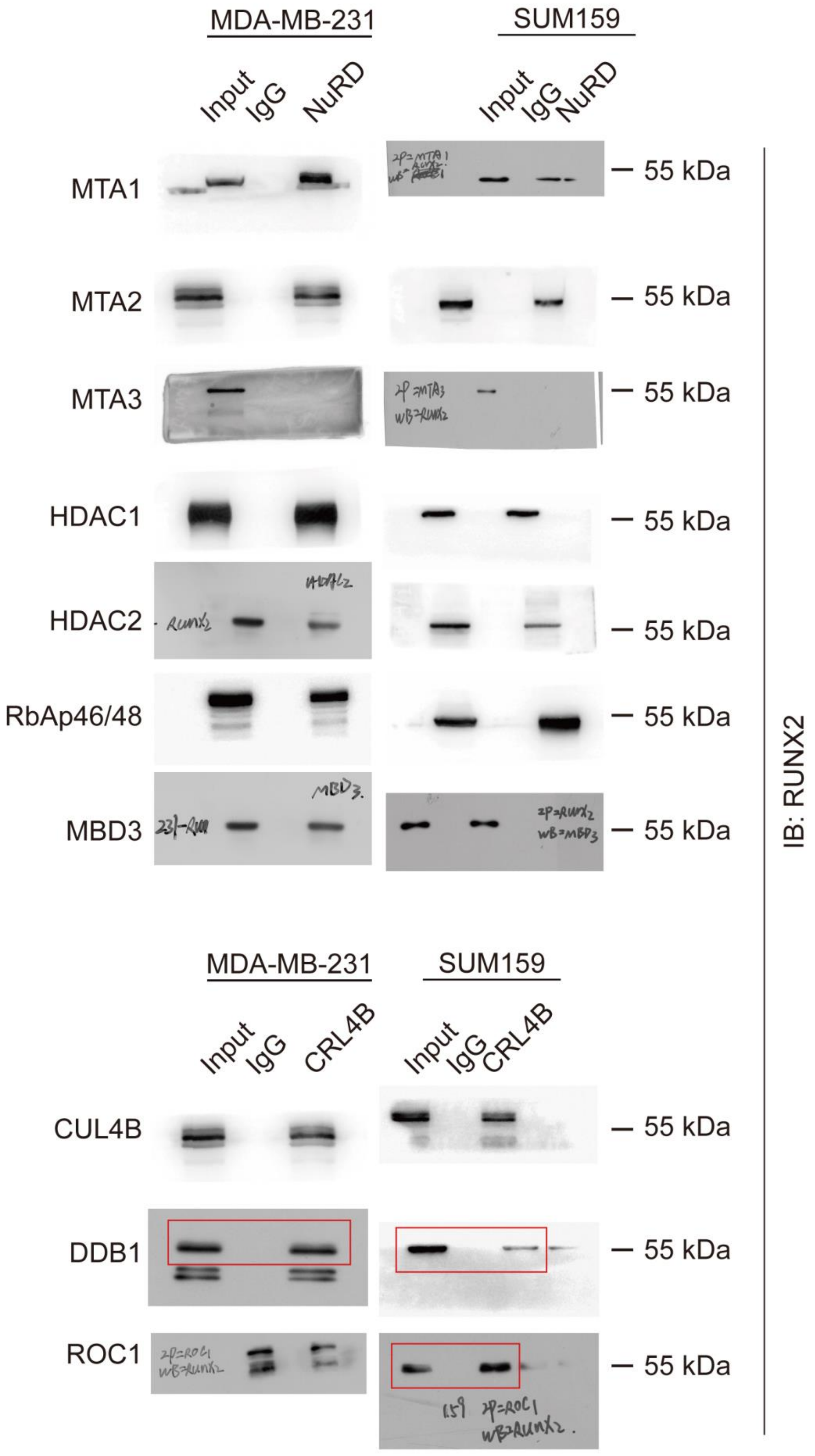

Uncropped blots related to Figure 2E-F

Figure 2E

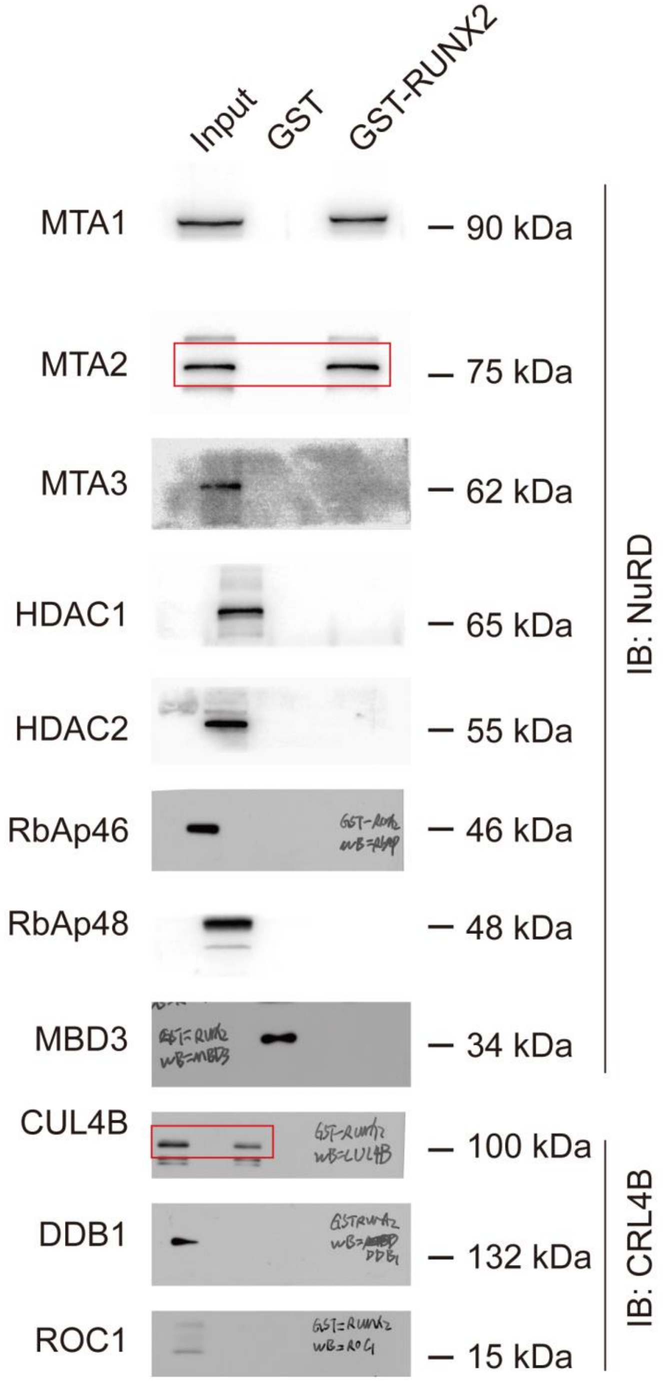

Figure 2F

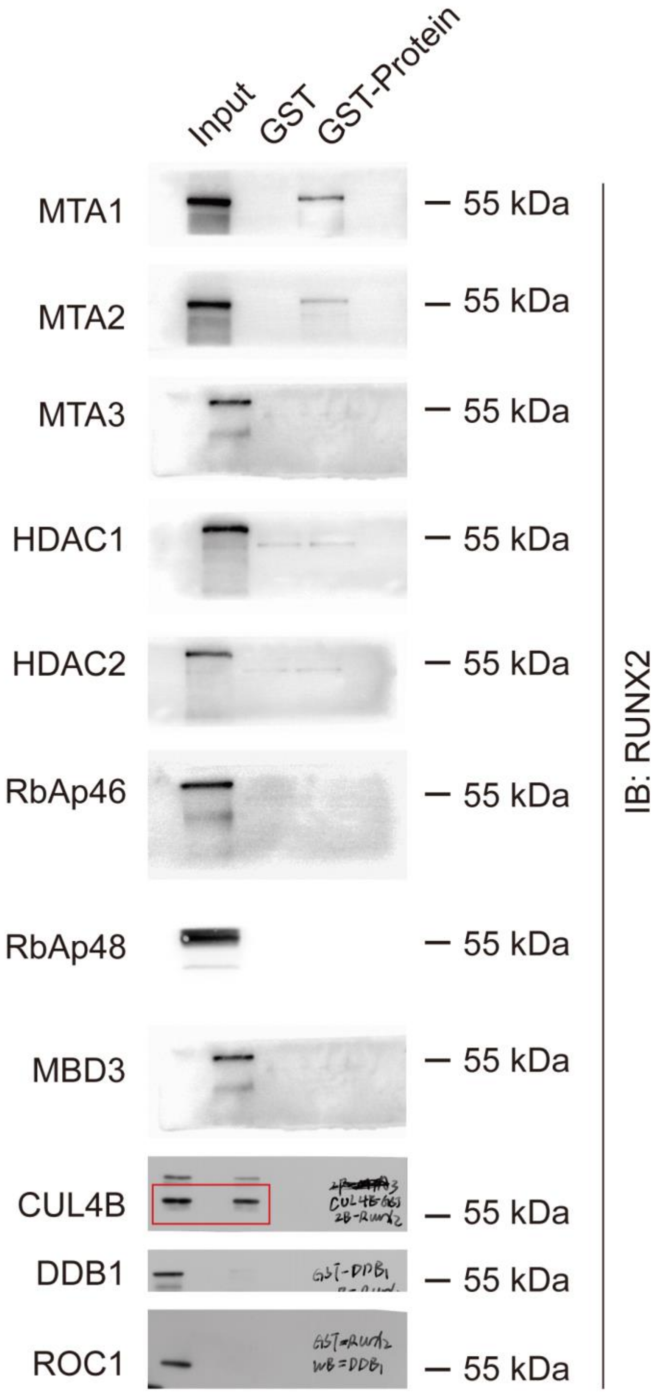

Uncropped blots related to Figure 2G-I

Figure 2G

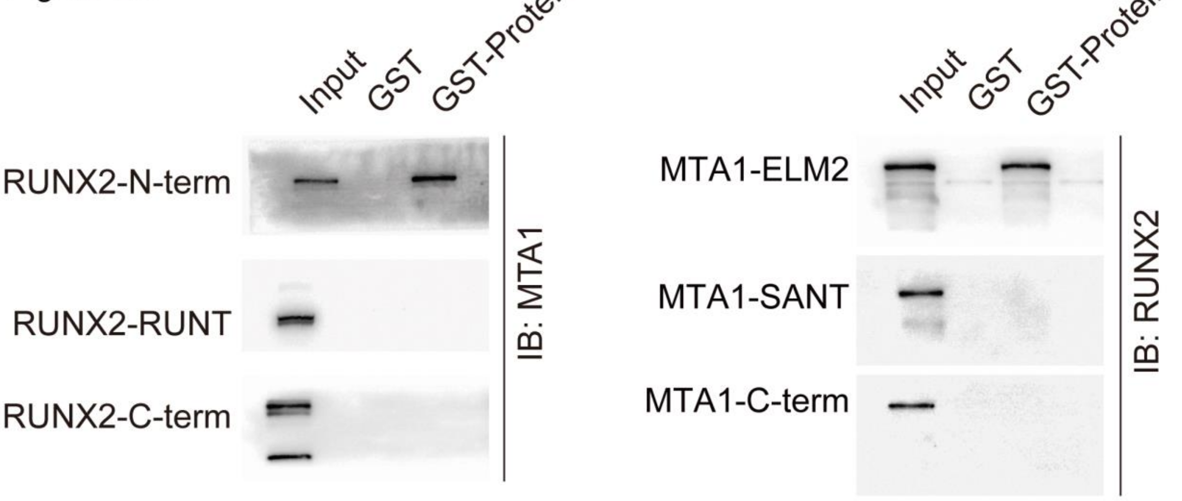

Figure 2H

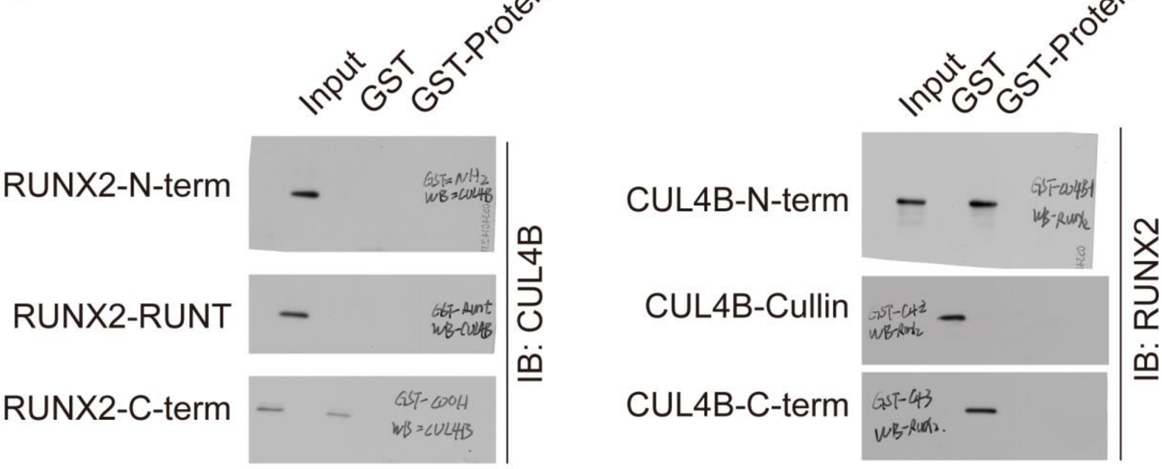

Figure 2I

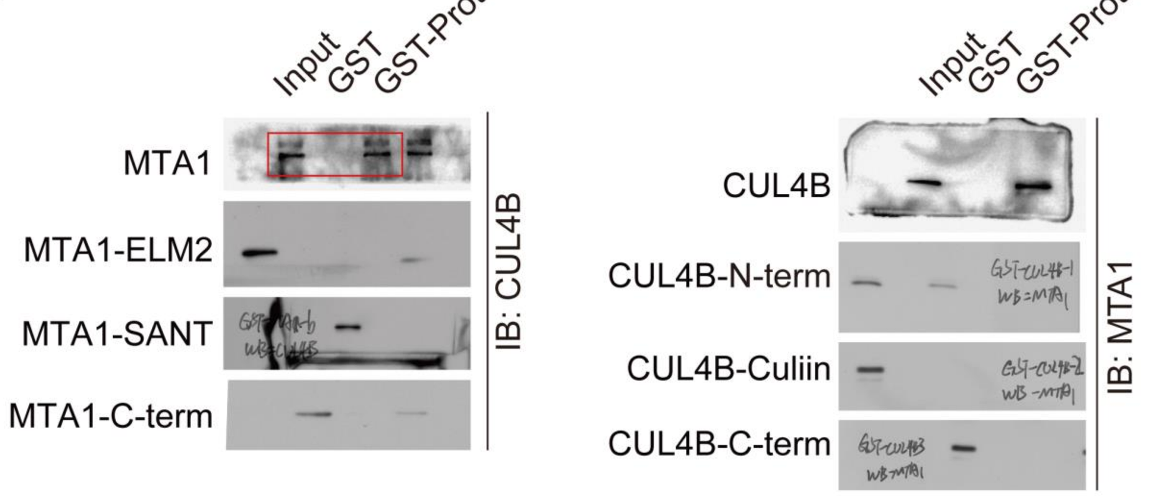

Uncropped blots related to Figure 3B

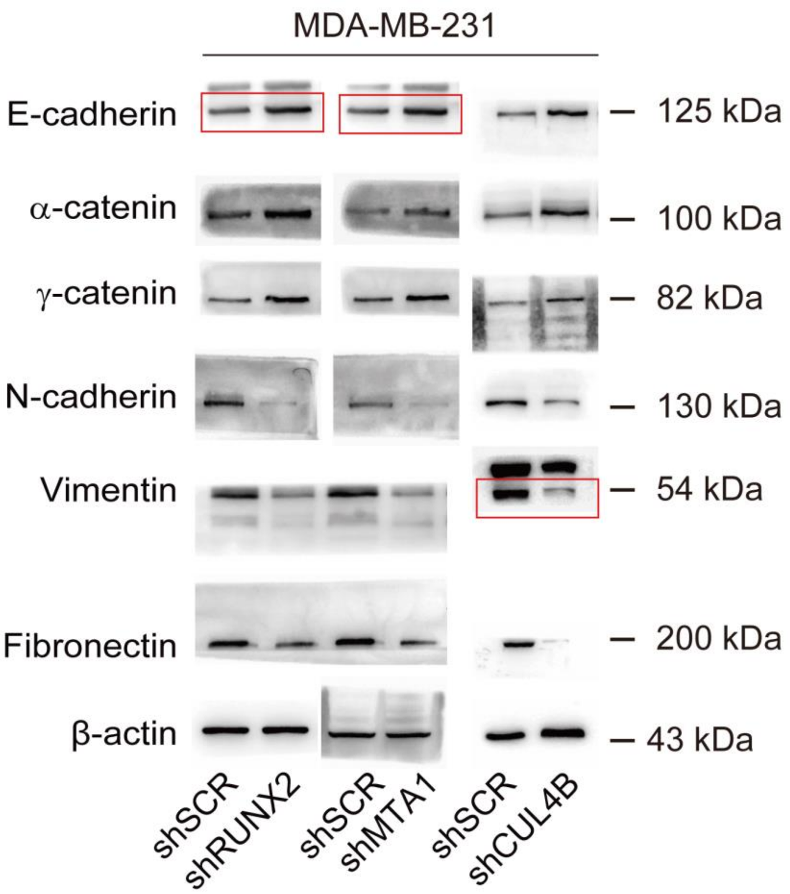

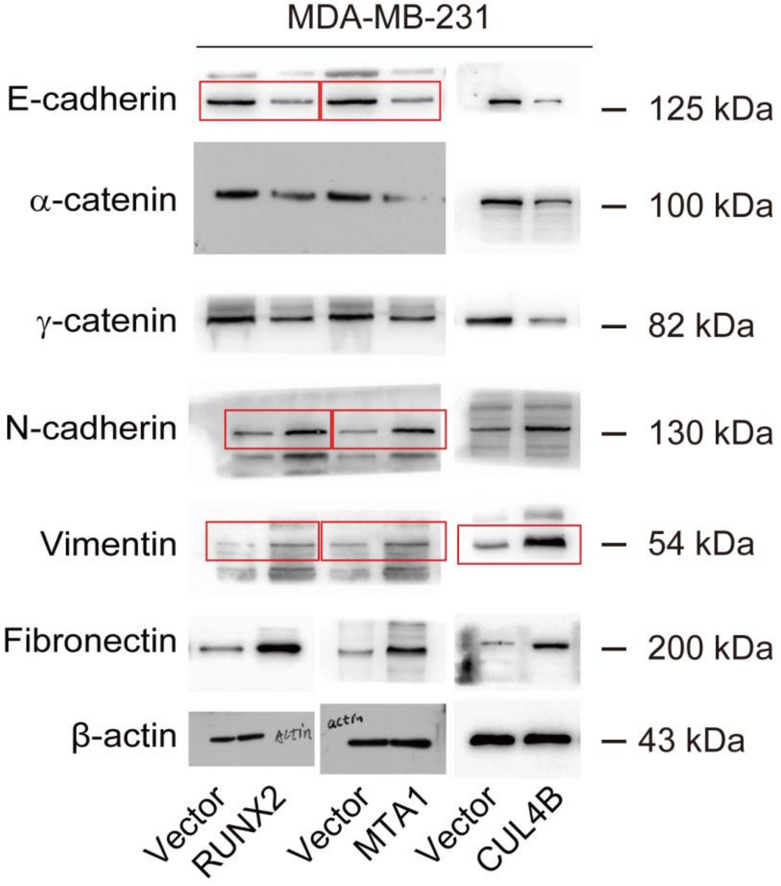

Uncropped blots related to Figure 3D

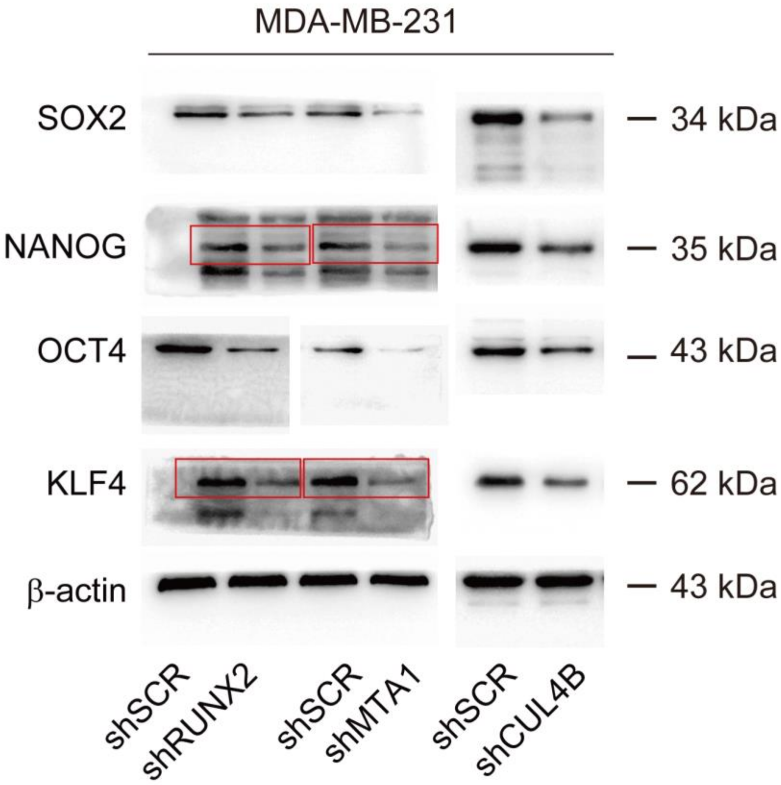

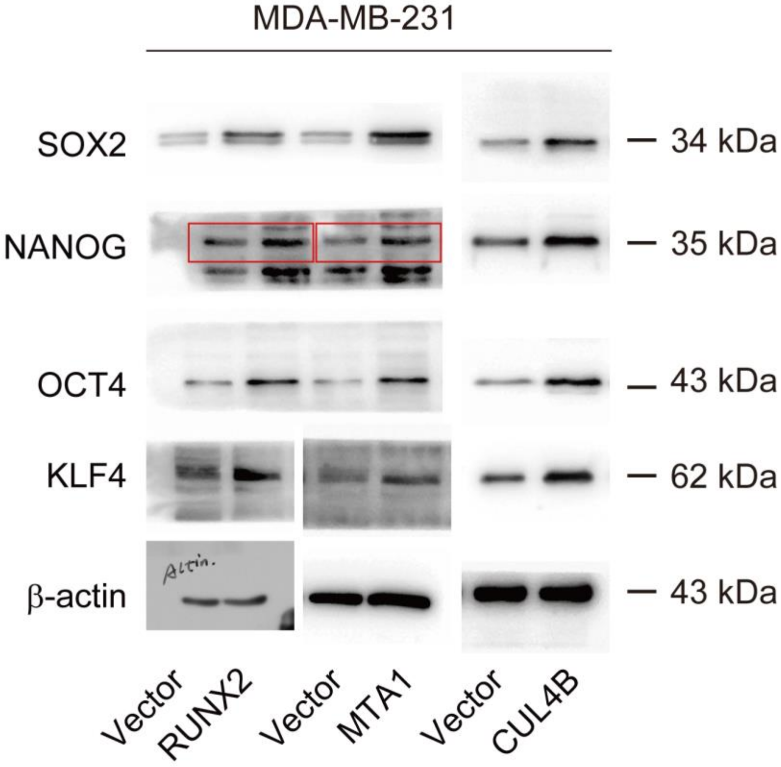

Uncropped blots related to Figure 3H

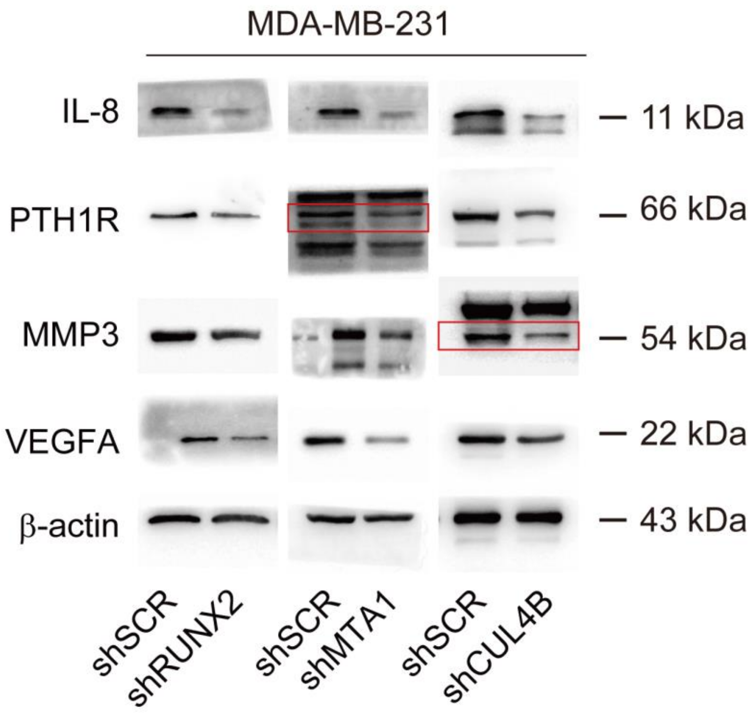

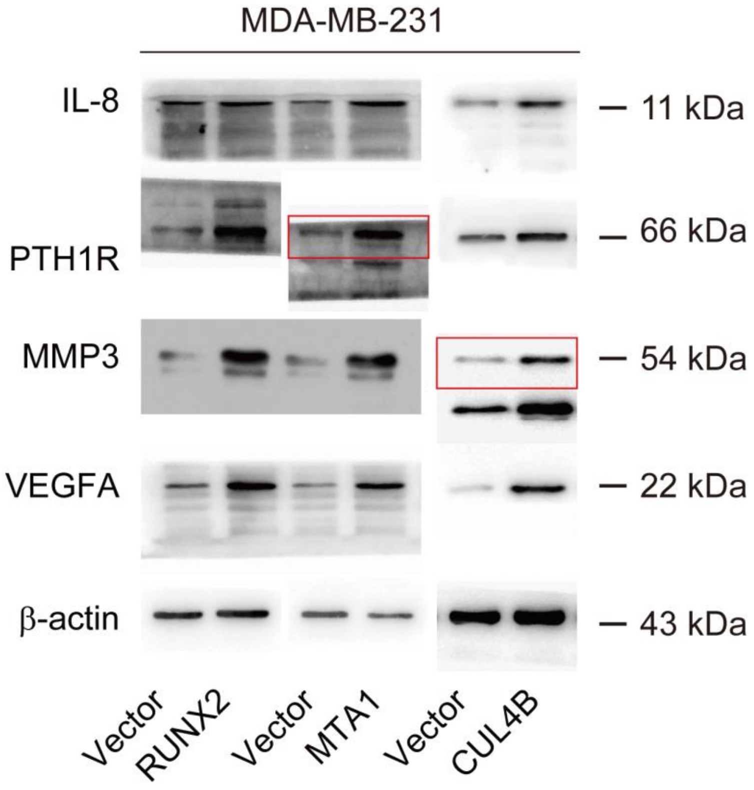

Uncropped blots related to Figure 4E

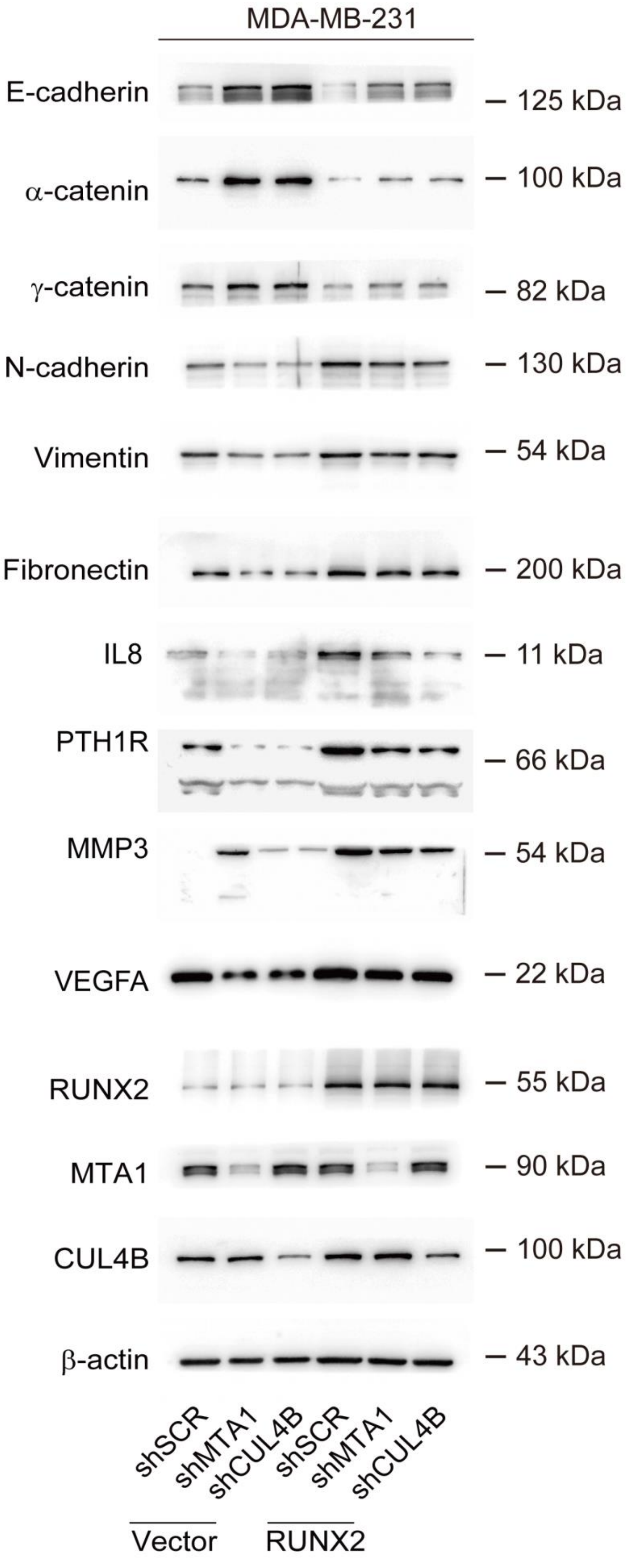

Uncropped blots related to Figure 5K-L

Figure 5K

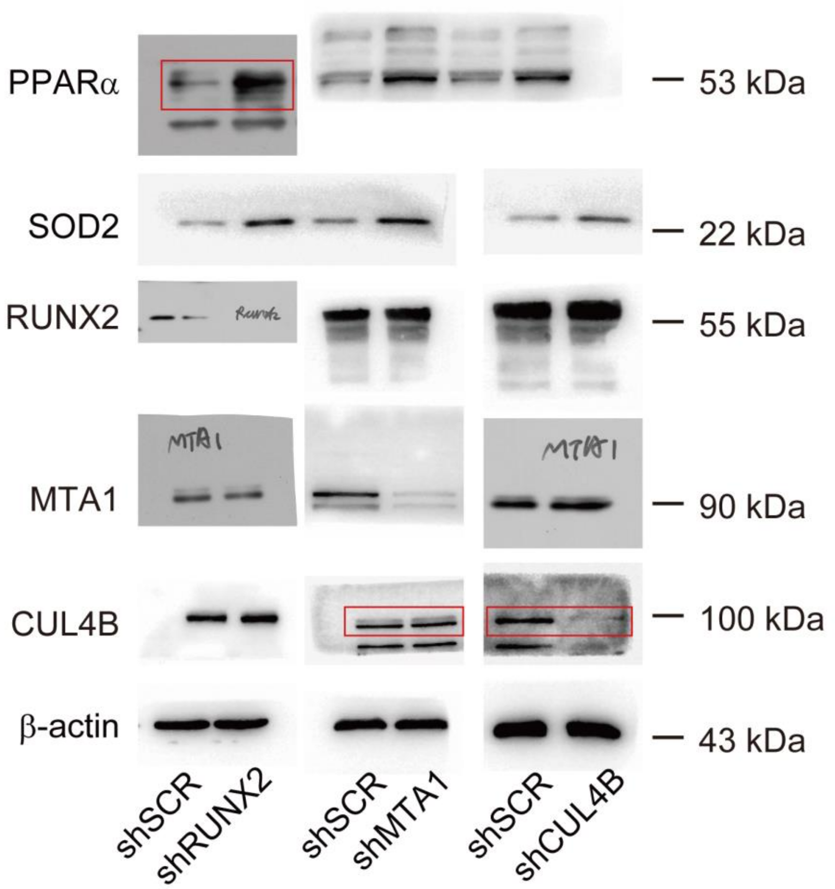

Figure 5L

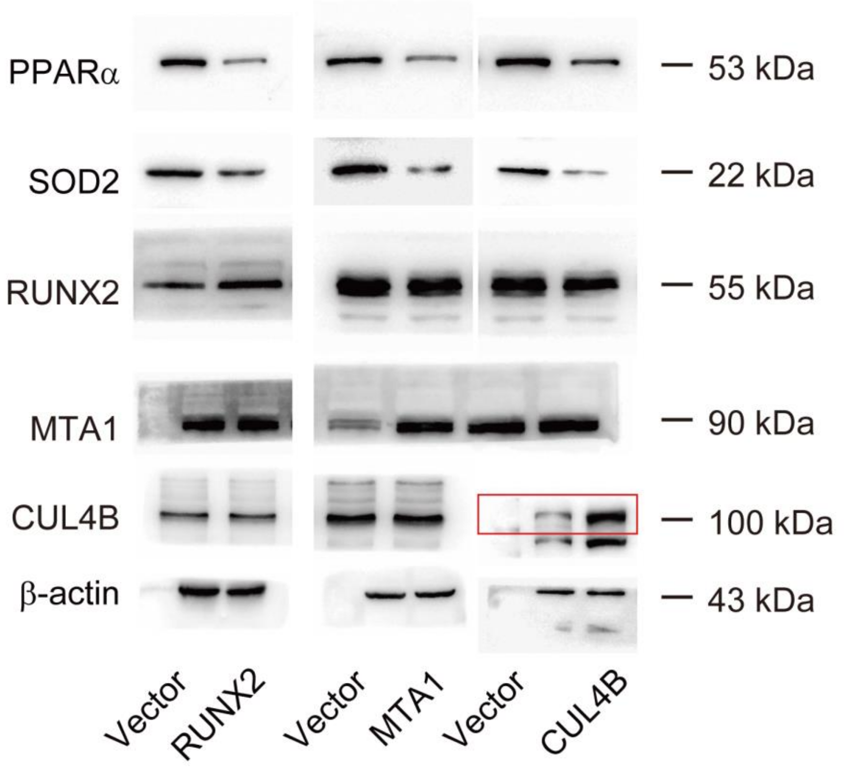

Uncropped blots related to Figure 6C

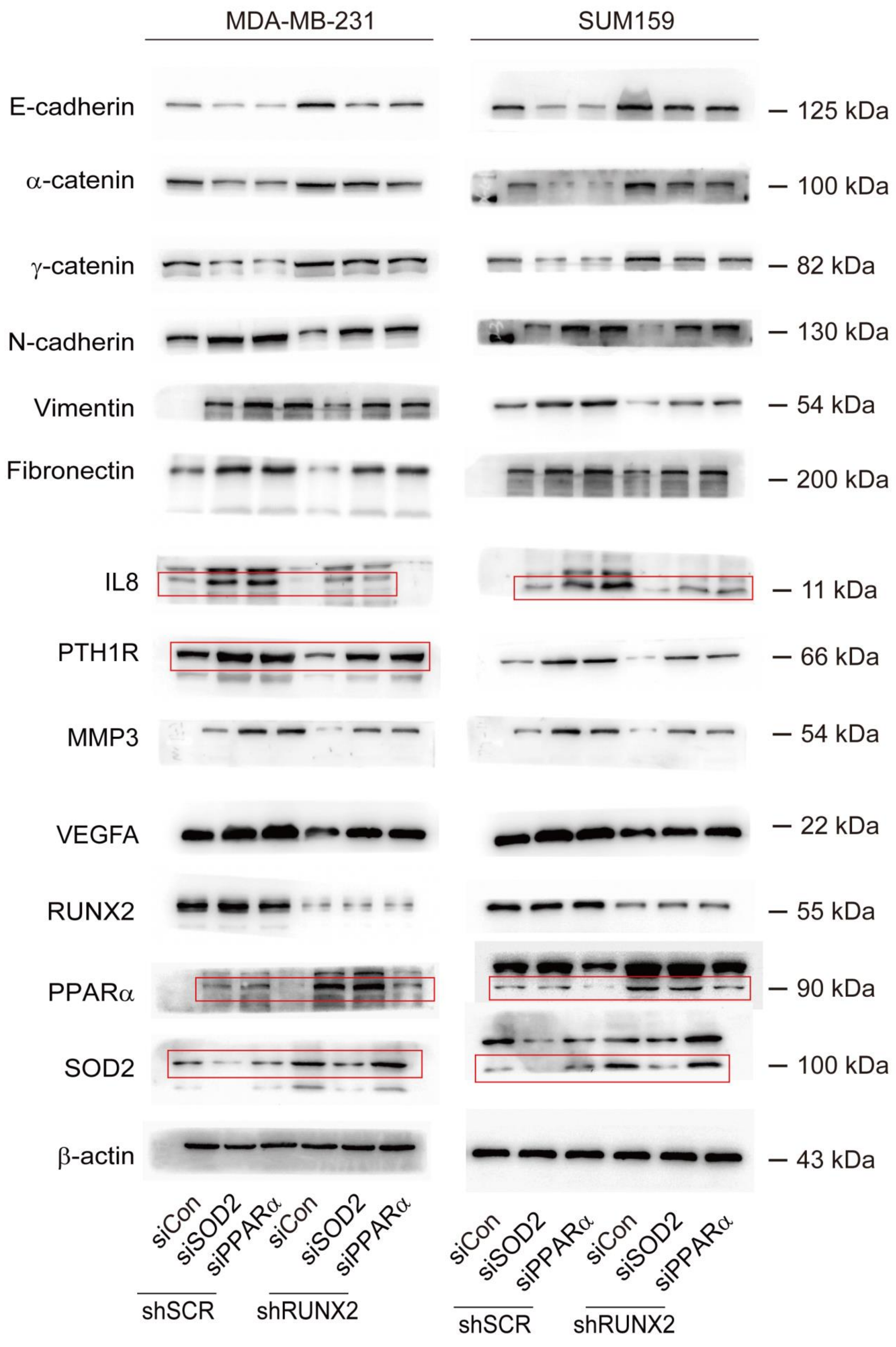

Uncropped blots related to Supplemental Figure 2A

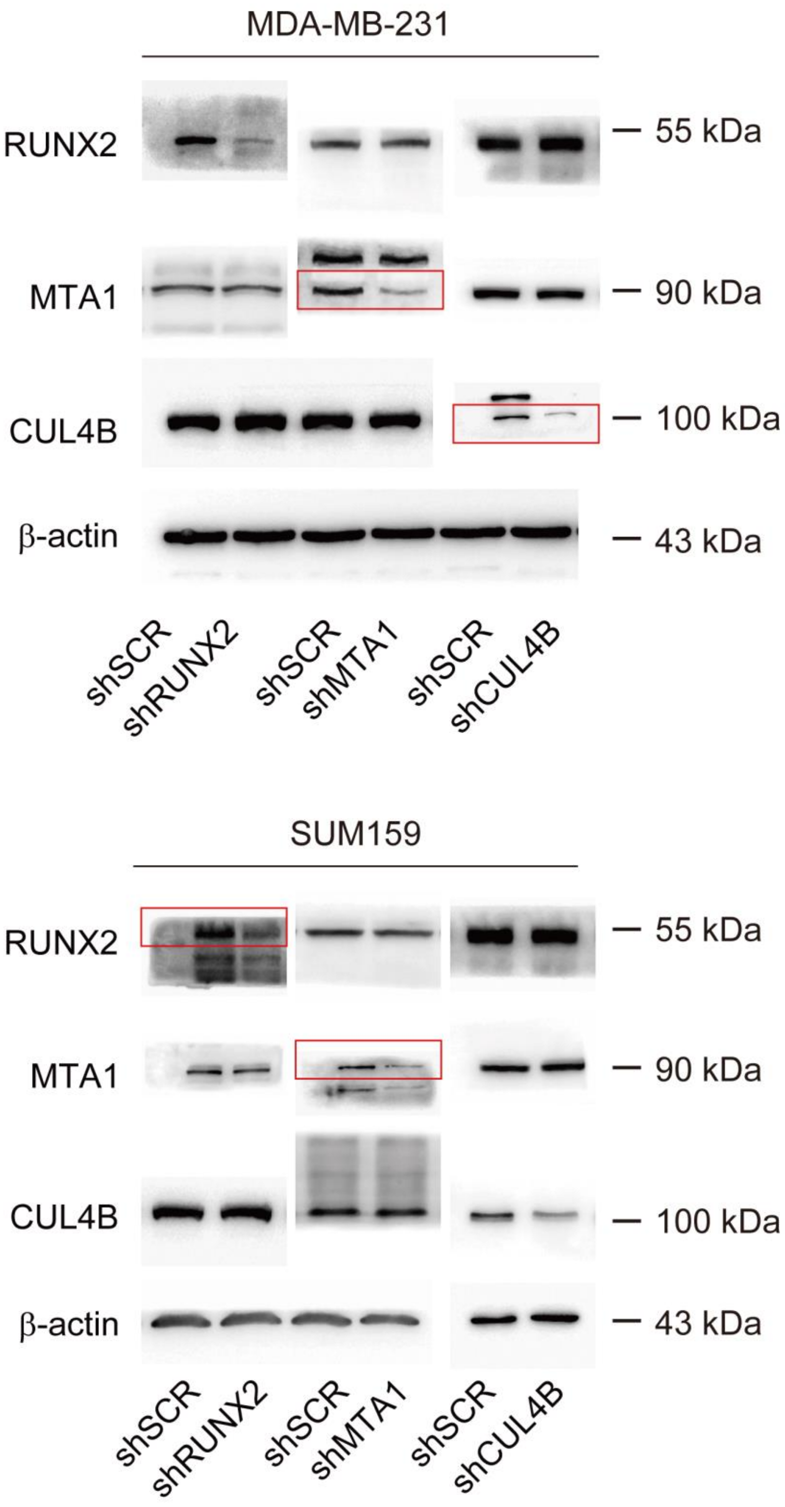

Uncropped blots related to Supplemental Figure 2B

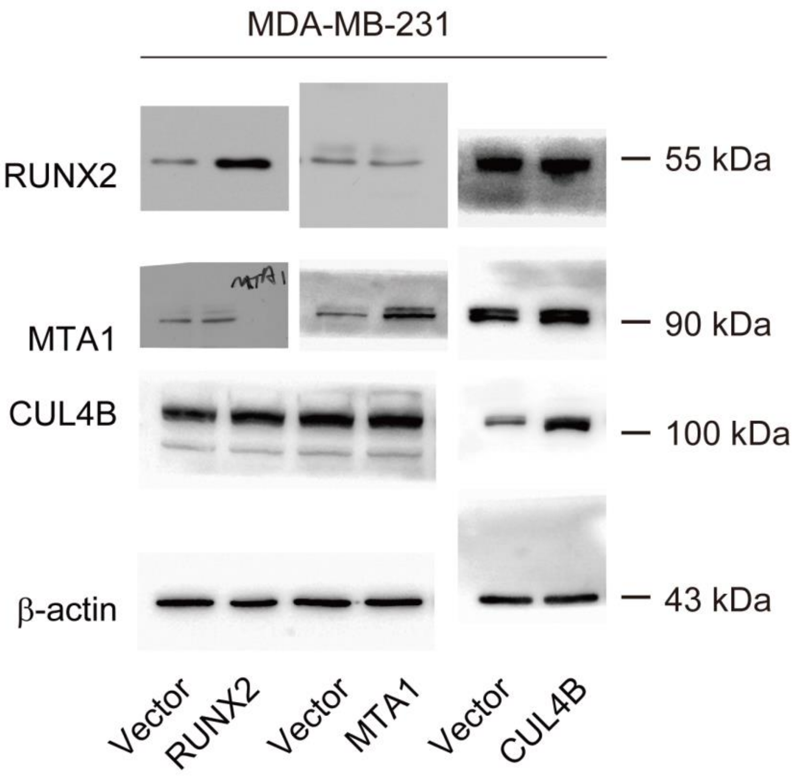

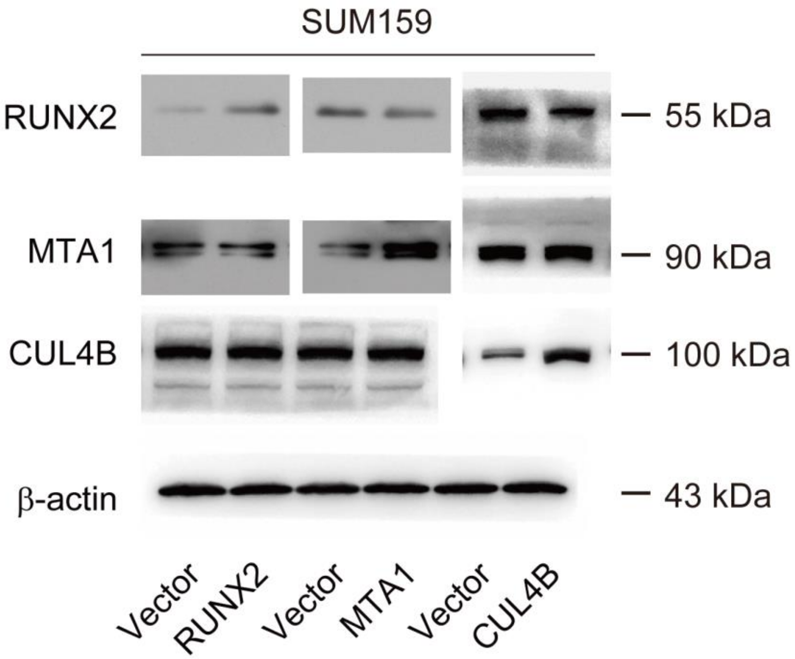

Uncropped blots related to Supplemental Figure 3B

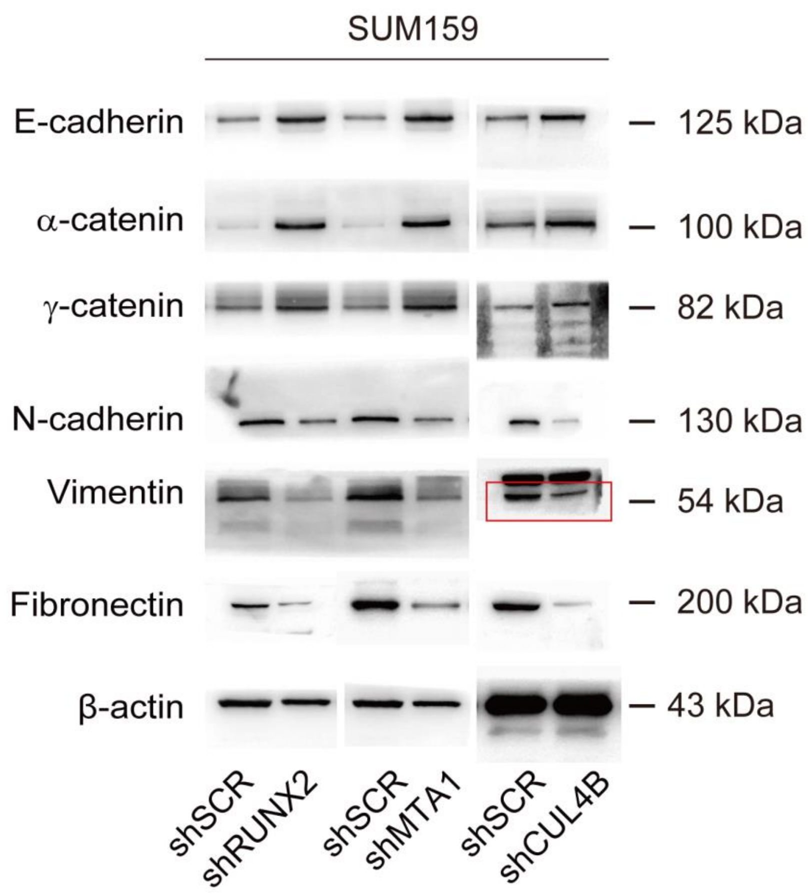

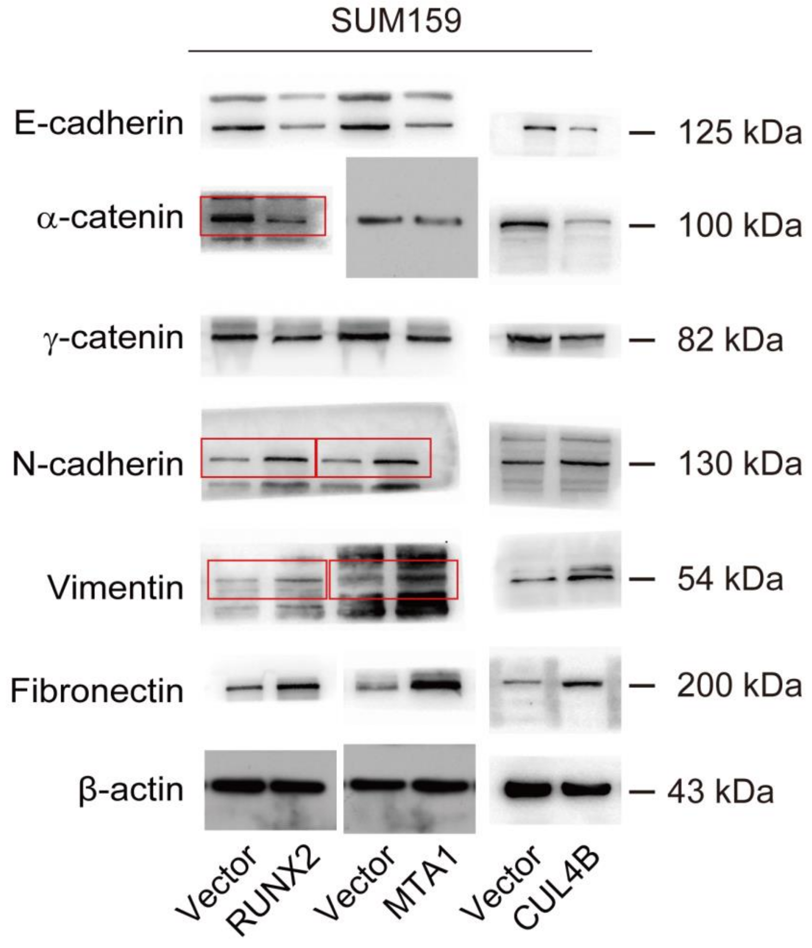

Uncropped blots related to Supplemental Figure 3D

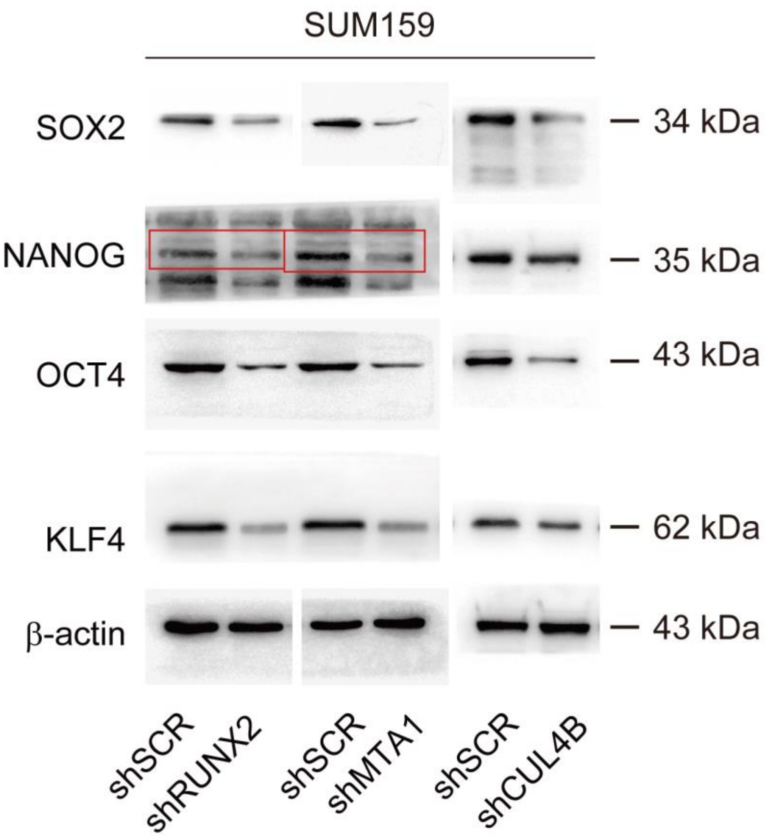

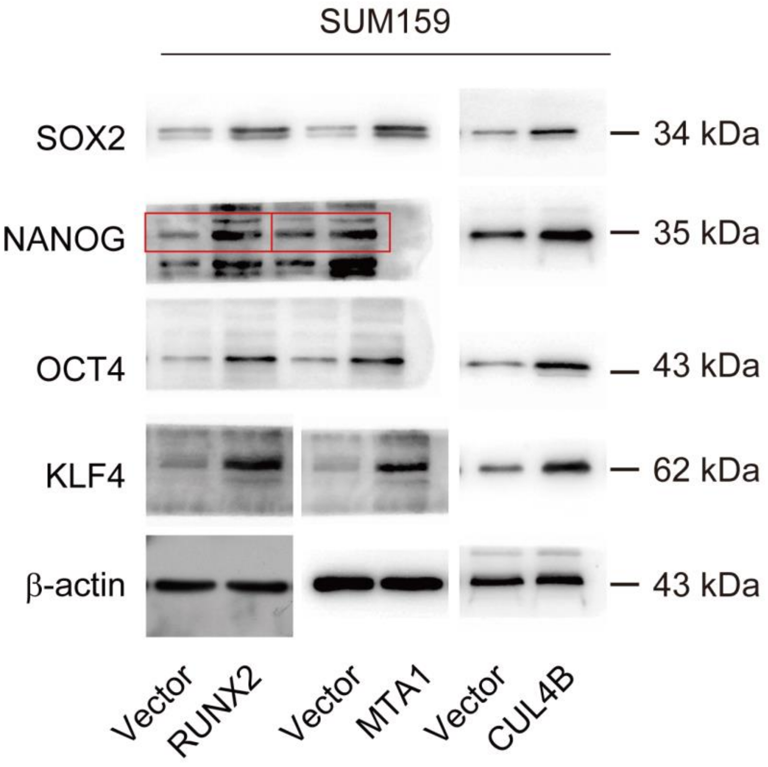

Uncropped blots related to Supplemental Figure 3H

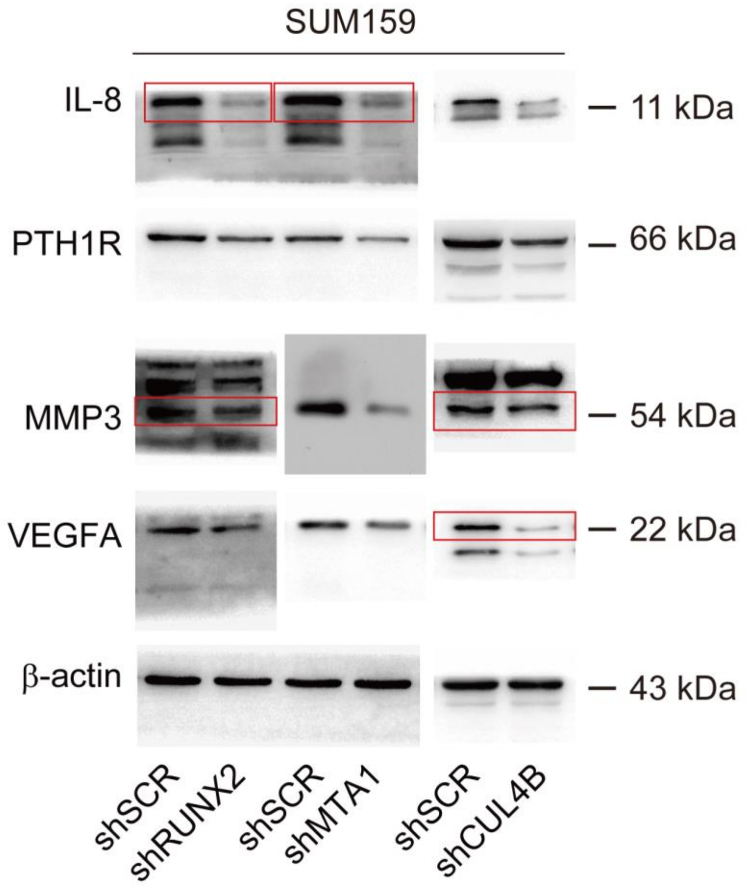

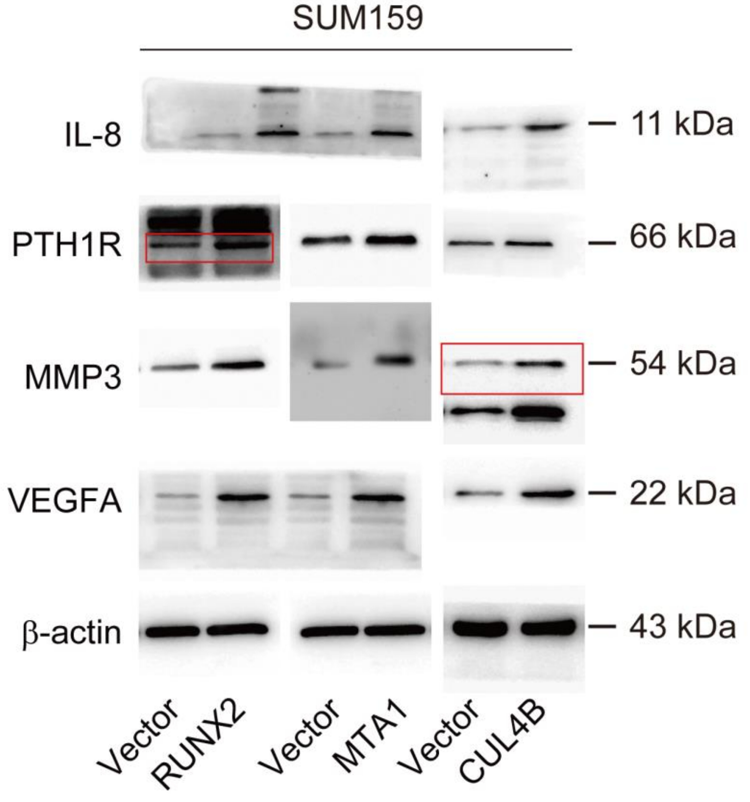

Uncropped blots related to Supplemental Figure 4B

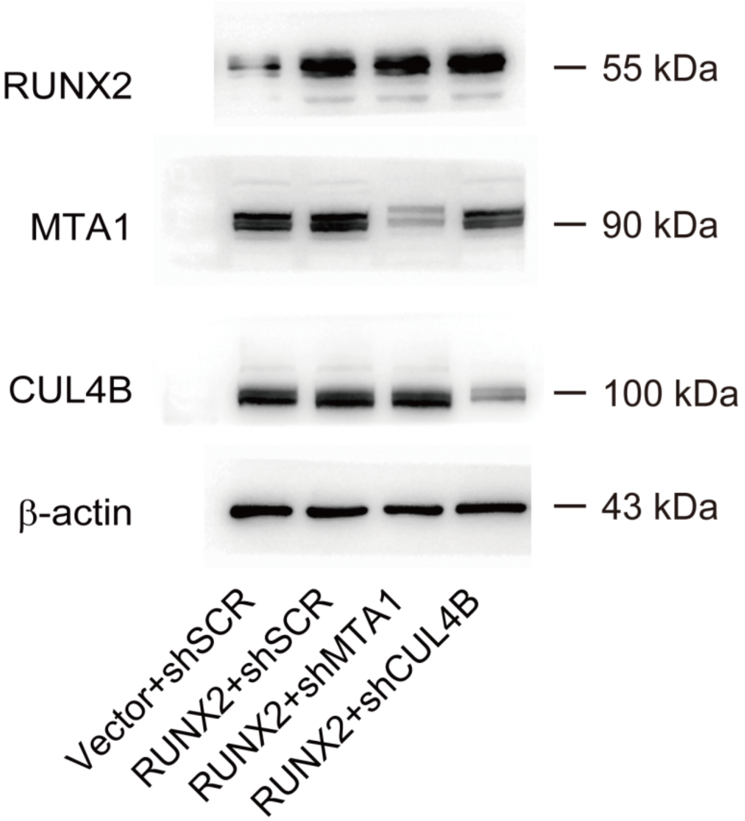

Uncropped blots related to Supplemental Figure 4E

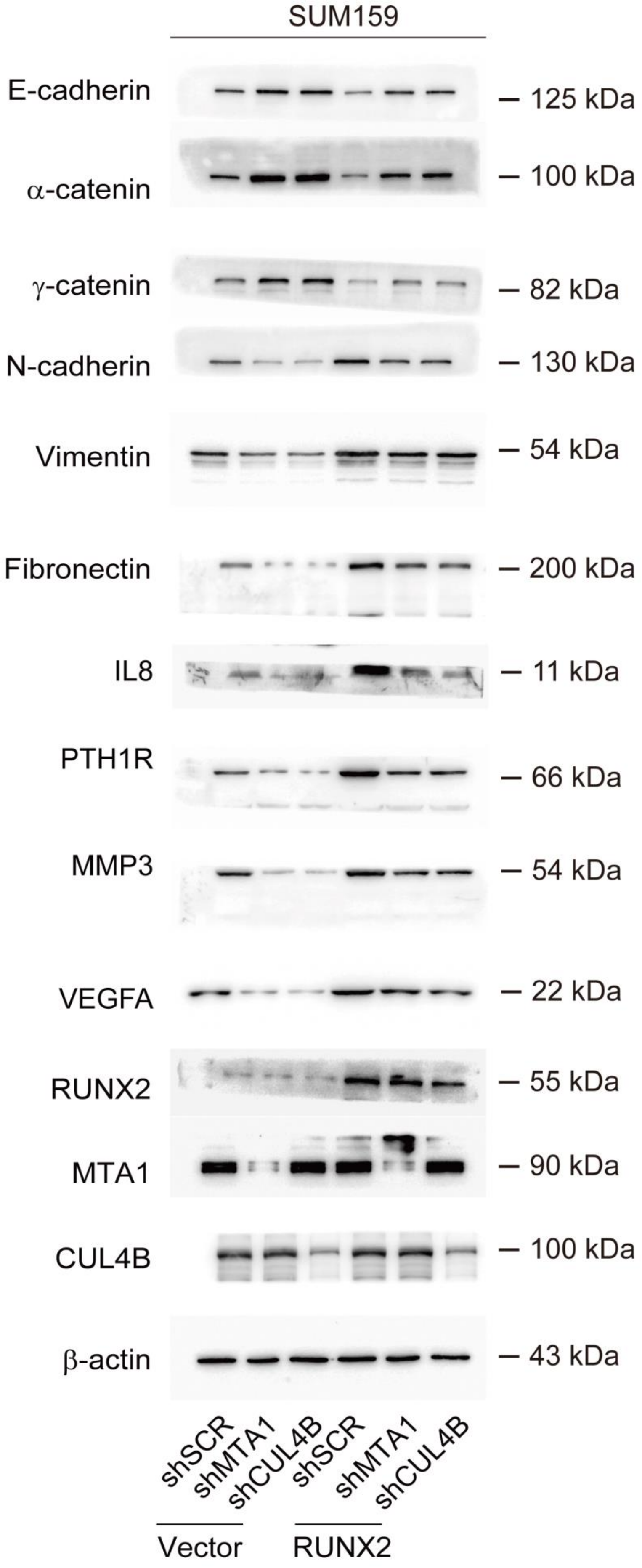

Supplement: Supplementary file 4 — Uncropped western blots related to results [file 41418_2022_1010_MOESM4_ESM.pdf]
